# Exploring the Chemical Complexity and Sources of Airborne Fine Particulate Matter in East Asia by Nontarget Analysis and Multivariate Modeling

**DOI:** 10.1021/acs.est.4c09615

**Published:** 2025-01-27

**Authors:** Jean Froment, Jong-Uk Park, Sang-Woo Kim, Yoonjin Cho, Soobin Choi, Young Hun Seo, Seungyun Baik, Ji Eun Lee, Jonathan W. Martin

**Affiliations:** † Department of Environmental Science, Stockholm University, Stockholm 10691, Sweden; ‡ Department of Environmental Chemistry and Health Effects, NILU, Kjeller 2027, Norway; § School of Earth and Environmental Sciences, Seoul National University, Seoul 08826, Republic of Korea; ∥ Energy & Environment Cluster, Planning and Coordination Division, Korea Institute of Science and Technology (KIST) Europe, Campus E 7.1, Saarbruecken 66123, Germany; ⊥ Chemical & Biological Integrative Research Center, Biomedical Research Division, Korea Institute of Science and Technology, Seoul 02792, Republic of Korea

**Keywords:** PM_2.5_, air pollution, high-resolution mass spectrometry, nontarget, sources, PFAS

## Abstract

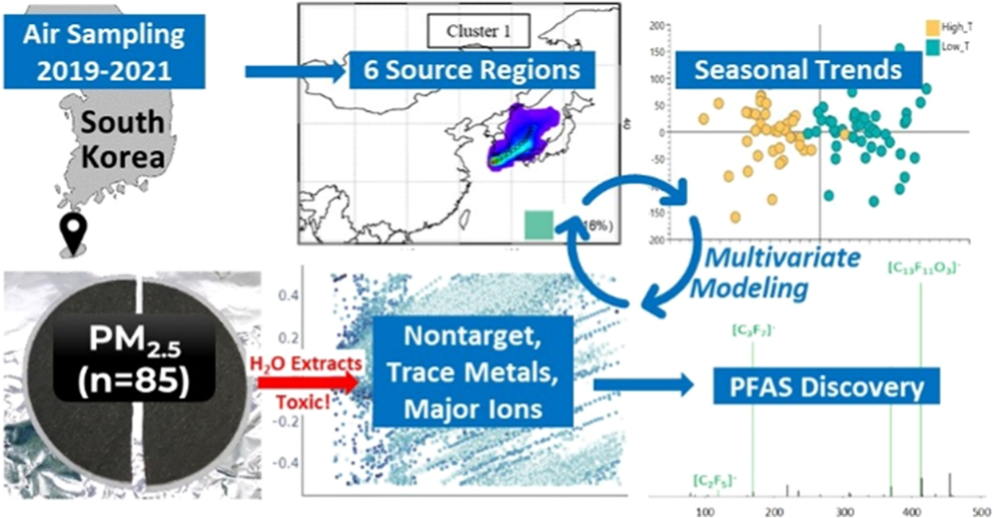

The complex and dynamic nature of airborne fine particulate matter (PM_2.5_) has hindered understanding of its chemical composition, sources, and toxic effects. In the first steps of a larger study, here, we aimed to elucidate relationships between source regions, ambient conditions, and the chemical composition in water extracts of PM_2.5_ samples (*n* = 85) collected over 16 months at an observatory in the Yellow Sea. In each extract, we quantified elements and major ions and profiled the complex mixtures of organic compounds by nontarget mass spectrometry. More than 50,000 nontarget features were detected, and by consensus of in silico tools, we assigned a molecular formula to 13,907 features. Oxygenated compounds were most prominent, followed by mixed nitrogenated/oxygenated compounds, organic sulfates, and sulfonates. Spectral matching enabled identification or structural annotation of 43 substances, and a workflow involving SIRIUS and MS-DIAL software enabled annotation of 74 unknown per- and polyfluoroalkyl substances with primary source regions in China and the Korean Peninsula. Multivariate modeling revealed seasonal variations in chemistry, attributable to the combination of warmer temperatures and maritime source regions in summer and to cooler temperatures and source regions of China in winter.

## Abstract 2

The water-soluble fraction of airborne fine particulates was comprehensively characterized over 16 months to better understand the chemical nature and sources of organic and inorganic substances contributing to air pollution in east Asian regions.

## Introduction

Exposure to airborne fine particulate matter (i.e., PM_2.5_; diameter <2.5 μm) has been linked to early death, cancer, stroke, and chronic lung and heart disease.^1,2^ PM_2.5_ can be deposited deep in the lungs, and the ultrafine fraction (i.e., <0.1 μm) can even cross biological barriers to enter the bloodstream,^3^ making PM_2.5_ a source of exposure to physical particulates and its entrained chemical load.^4^ A large fraction of PM_2.5_ by mass is highly water-soluble, an important factor affecting exposure through inhalation.^4^ Although a global problem, some of the highest levels of PM_2.5_ are reported in regions of the Asian continent.^5^ Prior to the global pandemic, in 2019, people in China and South Korea were exposed to PM_2.5_ levels exceeding the World Health Organization’s air quality guideline limit (15 μg/m^3^) on most days of the year (336 days in China, 319 days in South Korea).^5^ Some prevention programs have been implemented; for example, in China, the Air Pollution Prevention and Control Action Plan helped to reduce PM_2.5_ between 2013 and 2017, highlighting the need for stronger sustainable efforts.^6^ Similar emission control policies in South Korea helped to reduce PM_2.5_ pollution by 9% between 2016 and 2020.^7^ Additionally, since 2020, a management system has been implemented to reduce seasonal emissions of PM_2.5_.^8^

The chemical composition of PM_2.5_ includes a complex and dynamic mixture of organic substances, as well as inorganic ions, trace elements, and black carbon.^9−11^ Advancements in high-resolution mass spectrometry (HRMS) methods are now allowing wider targeted analysis and nontarget profiling for identification and broad molecular characterization of organic substances in PM_2.5_.^9,12−17^ In previous work, we applied nontarget HRMS to PM_2.5_ emitted from regions of South Asia and demonstrated that the complexity of organic substances was greatest in the water-soluble fraction, compared to polar and nonpolar solvent extracts.^14^ The water-soluble fraction of PM_2.5_ has been identified as a contributor to toxicity, including as the main contributor to reactive oxygen species generation in rat cells^18^ and in the acellular dithiothreitol (DTT) assay, where the important role of water-soluble organic compounds (WSOCs) has also been demonstrated.^19^ Moreover, in aqueous extracts of PM_2.5_ from China, Di et al. observed a correlation between the total concentration of WSOCs and apoptosis in exposed human lung cells.^20^ The ecotoxicity of the water-soluble fraction of PM_2.5_ is also relevant, given its demonstrated toxicity to green algae.^21^ Compared to hydrophobic substances, water-soluble components of PM_2.5_ are more likely to be bioavailable and interact rapidly with biological systems when inhaled,^4,22^ and a previous study has shown that the water-soluble fraction is also the most chemically diverse.^14^ However, the limited number of studies characterizing WSOCs from PM_2.5_ poses a challenge to understanding its chemical composition and toxicity. Therefore, this study proposes to characterize both inorganic and organic components, moving beyond the suspect screening of WSOCs, and aims to understand the critical changes in water-extracted PM_2.5_ depending on the sources and atmospheric conditions.

For the present study, we collected PM_2.5_ by a high-volume air sampler (48–72 h) between November 2019 and March 2021 (a total of 85 samples) at the Gosan Climate Observatory (Jeju Island, Republic of Korea). Aqueous extracts of PM_2.5_ are cytotoxic to primary human cells. Thus, the primary study objective was to comprehensively characterize inorganic and organic substances in each of these toxicologically relevant water-soluble fractions. A series of chemical analyses were conducted on each extract by employing nontarget HRMS for organic substances, inductively coupled plasma (ICP)-MS for metals and metalloids, and ion chromatography for major ions. A secondary objective was to use multivariate tools to understand how the complex chemical compositions changed with the back-trajectory source and atmospheric conditions. Overall, the chemical complexity and seasonal variability of these toxic samples are presented; hazardous anthropogenic pollutants are discovered; and associations between PM_2.5_ chemistry, meteorology, and air–mass origins are elucidated. These results will inform future work on the molecular drivers of toxicity in PM_2.5_.

## Materials and Methods

### Sample Collection and Handling

Ambient monitoring and sample collection were carried out at the Gosan Climate Observatory (33.2933°N, 126.1625°E, 72 m above mean sea level), located in the Yellow Sea on Jeju Island, Republic of Korea. Due to its location in the path of aerosols transported from the Asian continent,^23^ it is a strategic receptor site for monitoring air pollution originating from China, Japan, and the Korean Peninsula.^10,24^ Sampling of airborne fine particulates was performed by using a high-volume sampler with a PM_2.5_ selective inlet (sampler model: DH-77, Digitel Elektronik AG, Volketswil, Switzerland). PM_2.5_ samples were collected on in-line quartz filters (150 mm ⌀) prepared from quartz fiber membranes (Merck Millipore, France) and prefurnaced at 450 °C for 6 h in individual aluminum envelopes. Filter presampling weights were recorded under controlled temperature (20 °C) and humidity (50%) after 24 h of equilibration. The filters were shipped in individually sealed aluminum envelopes and sealed bags to the observatory. Each filter was exposed for 48–72 h at a calibrated flow of 500 L/min, typically three filters per week, plus a field blank. Samples were kept frozen (−20 °C) and shipped to the laboratory in Stockholm, where postweights were recorded in the same climate-controlled room as preweights. In total, 102 filters were collected, but due to high winds and rain during collection, 15 filters were noted to be wet during collection and were therefore excluded, leaving 85 PM_2.5_ samples for analysis here, along with 30 field blanks.

### Back-Trajectory Calculations and Clustering

**Figure 1:**
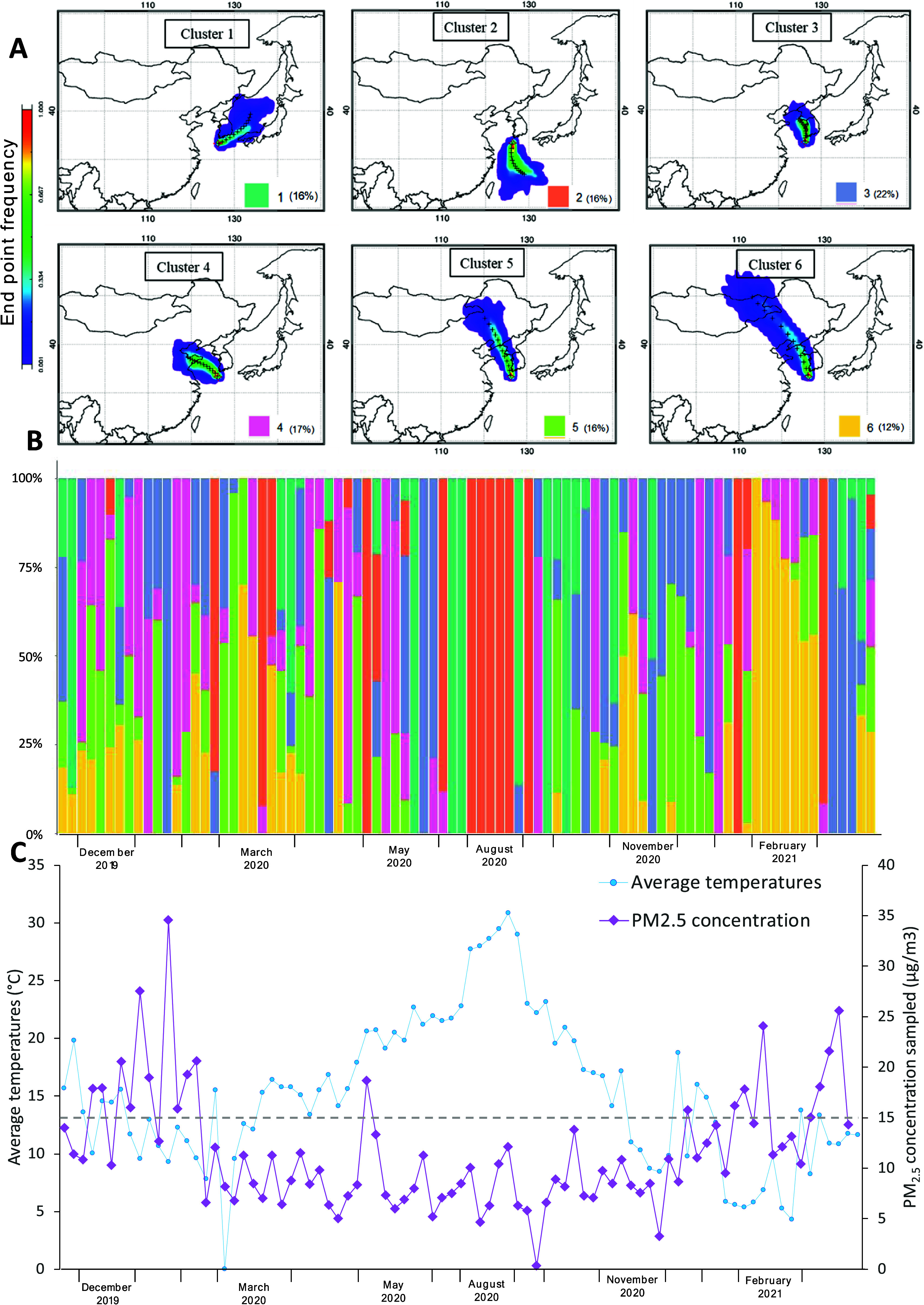
(A) Over the campaign, backward trajectories (48 h) of sampled air calculated every hour clustered to six source regions: 1—East Sea/Sea of Japan; 2—East China Sea; 3—Korean Peninsula; 4—Shandong/Beijing; 5—North China, 6—North China/Mongolia. For each sample (*n* = 85), the (B) relative contribution (%) of air coming from each source region and (C) average air temperatures (°C) and PM_2.5_ concentrations (μg/m^3^, gravimetric on filters) are shown. The WHO air quality guideline limit is also shown as a dashed line (15 μg/m^3^). The time axes of (B) and (C) are not continuous due to missing or excluded samples because of storms.

For the sampling period of each sample, 48 h back-trajectories of the air reaching the observatory were calculated every hour using the Hybrid Single-Particle Lagrangian Integrated Trajectory model (HYSPLIT, v. 5.2.0^25^) with 3 h data from the National Centers for Environmental Prediction (NCEP) Global Data Assimilation System (GDAS) as input meteorological fields.^26^ The Fuzzy c-mean clustering method^27^ was used to classify the air–mass trajectories into six source regions, chosen as the optimal clustering result according to criteria outlined previously,^28,29^ resulting in 54.5% of back-trajectories being clustered to one of the six source regions. Each source region was numbered and named according to air–mass origins (Figure 1A): 1—East Sea/Sea of Japan, 2—East China Sea, 3—Korean Peninsula, 4—Shandong/Beijing, 5—North China, and 6—North China/Mongolia.

### Aqueous Extraction of PM_2.5_

From all filters in the sampling period, WSOCs were extracted using the same protocol as Papazian et al.^14^ Briefly, half of each filter was cut using clean scissors and tweezers, weighed in a controlled climate room, and placed in a centrifuge tube (high-grade γ-resistant polypropylene, VWR International AB, Sweden) for extraction with 20 mL of high-performance liquid chromatography (HPLC)-grade water (Honeywell AB, Bromma, Sweden). The tubes were then sonicated at room temperature for 30 min and centrifuged for 10 min at 1800*g*. The resulting supernatant was recovered and filtered with 0.2 μm cellulose syringe filters (Minisart RC 15 mm, Sartorius AG, Göttingen, Germany), and eight internal standards were spiked in all of the extracts at 0.23 μg/mL each (phenylglyoxylic acid-*d*_5_, daidzein-*d*_6_, *N*-acetyl-*d*_3_-*S*-(*N*-methylcarbamoyl)-l-cysteine, atrazine-2-hydroxy-*d*_3_, norharmane-*d*_7_, atrazine-*d*_5_, 4-(methylnitrosamino)-1-(3-pyridyl)-1-butanol-*d*_5_, and racemic enterolactone-^13^C_3_). The internal standards were not added before extraction because a portion of each extract was used for toxicity tests. The aqueous extracts were stored at −20 °C before chemical and toxicological analyses.

### Nontarget HRMS Analysis

The water extracts were analyzed by in-line solid-phase extraction (SPE)–liquid chromatography (LC)–HRMS, following the same protocol and instrumental setup as Papazian et al.^14^ Briefly, 1.05 mL of aqueous extracts was injected into two in-line SPE columns in series: polar end-capped C18 (Thermo Scientific Hypersil Gold aQ, 2.1 mm × 20 mm, 12 μm particle) followed by pentafluorophenyl (Thermo Scientific Hypersil Gold PFP, 2.1 mm × 20 mm, 12 μm particle). The SPE columns were then reverse-flow-eluted by the mobile-phase gradient onto an analytical C18 UPLC column (Waters Acquity UPLC BEH C18, 3.0 × 100 mm^2^, 1.7 μm particle) with a 0.2 μm in-line filter in a column oven at 40 °C. The gradient flow was 0.4 mL/min and composed of water containing 0.1% formic acid (mobile phase A) and methanol containing 0.1% formic acid (mobile phase B). The initial conditions were 5% B, increasing to 95% after 24 min before returning to initial conditions for 4 min of equilibration.

The chromatographic setup was coupled to a quadrupole orbitrap mass spectrometer (Q Exactive Orbitrap HF-X, Thermo Fisher) and operated in electrospray ionization (ESI) mode, with separate injections performed for positive and negative ESI modes. The capillary voltage was +3.0 or −2.5 kV, the capillary temperature was 300 °C, and the auxiliary gas temperature was 400 °C. The sheath gas flow was 50, the auxiliary gas flow was 16, and the sweep gas flow was 2 (arbitrary units). Spectral acquisition included full-scan (MS1, 90–1000 *m*/*z*) and data-independent (MS2 DIA) MS/MS acquisition with four isolation windows (*m*/*z* 90–320, *m*/*z* 315–551, *m*/*z* 542–778, and *m*/*z* 769–1000) and dual collision energies (20, 70 V). MS2 fragments from both collision energies were analyzed simultaneously in the orbitrap with an auto-gain control target of 10^6^.

Samples were injected in random order, instrument blanks (HPLC grade water) were injected after six sample injections, and a quality control (QC) sample was also created by pooling 32 randomly selected extracts, injected at the beginning and end of the injection sequence for both ionization modes. A retention time index (RTI) solution developed for nontarget screening^30,31^ was also injected on each day to aid structural annotations. Mass calibration was performed externally prior to each batch run (all of the injections were divided in seven batches) using the Pierce ion calibration solution (Thermo Scientific) for each mode.

### HRMS Data Processing

Software MS-DIAL (v. 3.78) was used to process raw data from nontarget HRMS acquisition,^32^ including for deconvolution of MS2 spectra. Injections of the RTI solutions were used to optimize MS-DIAL parameters, and the detected reference peaks in each mode (13 and 14, in positive and negative ionization modes, respectively) were manually checked to ensure that the peaks were correctly integrated and aligned and that MS2 spectra were accurately deconvoluted before proceeding (see the optimized parameters in Table S1). The field blanks were set as “blank” in MS-DIAL, and nontarget features in samples were only kept if their peak intensity was >5 times the field blank average. To correct all nontarget feature peak areas for instrumental drift or batch variation, integrated peak areas of the internal standards in all samples and field blanks were examined by principal component analysis (PCA), with unit variance no centering (UVN) scaling (SIMCA 17, Sartorius Stedim Data Analytics AB, Sweden). For results in each ionization mode, all peak areas in each sample were then normalized to internal standards by dividing peak areas with the corresponding PCA *t*1 score.^33^ To evaluate the impact of the back-trajectories on a feature’s intensity, the peak areas of the features were normalized between 0 and 1 throughout the data set, and the scaled area of the feature in all samples was multiplied by the source region cluster weight on the corresponding sampling days. For any chemical or nontarget feature, the relative contribution (%) of each source region cluster throughout the campaign was estimated by summing the peak area and the corresponding back-trajectory cluster weights for each sample, dividing it by the sum of all peak areas in all samples, and multiplying it by the cluster weight of the feature.

### Molecular Formula Assignments

The .mat files for all of the detected features were exported from MS-DIAL. The .mat format is compatible with other software and contains MS1 data, isotopic ratios, and fragmentation spectra of each nontarget feature. Molecular formula prediction of unknown features was performed using both SIRIUS (v. 5.6.3)^34^ and MS-FINDER (v. 3.52) software.^35^ Parameters used in SIRIUS were as follows: instrument, Orbitrap; MS2 mass accuracy, 3 ppm; isotopic score, SCORE; and database formula sources, NORMAN and PubChem. CSI:FingerID (which looks for structural fingerprints^27^) was enabled in SIRIUS to aid in ranking the predicted molecular formulas.^28^ Parameters used in MS-FINDER were the following: mass tolerance (ms1 and ms2), 3 ppm; formula finder checks, LEWIS and SENIOR rules and element probability check; element selection, O, N, P, S, F, Cl, Br, and I; data sources, T3DB and STOFF (environment); and use PubChem online when no query matches are found from a local database. The molecular formulas ranked first and second by both software were exported. When agreement between the two software tools was found, defined here as the same formula suggested by both *in silico* fragmentation tools within their top two candidates, the molecular formula was assigned. For further quality control, if the assigned formula contained Cl, the isotope ratio was manually checked, and the formula was only retained if the M + 2 isotope was also present.

### High-Resolution MS2 Spectral Library Searches

MassBank Europe and MassBank of North America (MoNA) were both downloaded in February 2021 (https://massbank.eu/MassBank/; https://mona.fiehnlab.ucdavis.edu/). The MSP files were used in MS-DIAL for spectral matching using an MS2 similarity score of at least 70%, MS1 tolerance of 0.01 Da, and MS2 tolerance of 0.015 Da. All initial annotations were then checked manually and discarded if they were deemed insufficient by visual inspection of the spectra. For the remaining annotated features, RTI was then calculated using the dedicated website at http://rti.chem.uoa.gr/. From the website, the equation given by the observed retention times of the RTI standards, injected with the samples, was retrieved and used to calculate feature RTIs (Figure S3). The predicted RTI of the candidate structure was retrieved from the online Norman Substance Database (https://www.norman-network.com/nds/susdat/susdatSearchShow.php). Finally, only those annotations with ΔRTI lower than or equal to 30 were kept, and the molecular structures were assigned with level 2 confidence according to the criteria of Schymanski et al.^36^ When possible, authentic standards were obtained and injected following the same instrumental setup, and their measured RTIs, precursor masses, isotopic ratios, and MS2 spectra were compared to the annotated feature to potentially confirm the structure at level 1 confidence.

### Analysis of Trace Metals and Transition Elements

An eighth of each sample filter was separately extracted in 10 mL of HPLC-grade water following the protocol described above, followed by sonication, centrifugation, and filtering. The new extracts were analyzed by inductively coupled plasma-mass spectrometry (ICP-MS; iCAP Qc, Thermo Fisher Scientific, Germany). Quantification was by calibration curves (Al, Ti, Cr, Mn, Fe, Co, Ni, Cu, Zn, Ga, Sr, Ag, Ca, Ba, Tl, Pb, and B) normalized to the internal standard response (In and Sc). All standards were Certipur-grade (Merck). Kinetic energy discrimination mode, using 99.999% helium gas, was applied to the selected compounds. Instrument detection limits were between 0.1 and 10 ppb.

### Analysis of Major Ions

Inorganic constituents were measured by ion chromatography (IC, Dionex Aquion, Thermo Finnigan LLC, Walkersville, United States) in the same aqueous extracts as for nontarget LC-HRMS. The 11 major ions routinely detected in PM_2.5_ were quantified by calibrations curves between 0.1 and 100 mg/L, including 6 anions (chloride, nitrite, bromide, nitrate, phosphate, sulfate) and 5 cations (sodium, ammonium, potassium, magnesium, calcium).^10,37^ As a quality control, two reference solutions with known concentrations of chloride, sulfate, sodium, and potassium (i.e., two anions and two cations) were also injected before the samples on each day of analysis. Signals of these reference samples were stable, with a relative standard deviation for all ions in the range of 0.2–1.5%. When necessary, the concentrations in sample extracts were corrected by subtracting the highest concentration detected in field blanks.

### Monitoring of Ambient Pollutants at the Gosan Climate Observatory

The ambient ground-level concentrations of gaseous pollutants (i.e., SO_2_, CO, O_3_, NO_2_) and the mass concentration of particulate matter (i.e., PM_2.5_, PM_10_) were measured simultaneously on an hourly basis by an air quality monitoring station (AQMS), processed under the framework of the National Ambient air quality Monitoring Information System (NAMIS) in South Korea.^23^ Hourly PM_10_ and PM_2.5_ mass concentrations were measured using the filter-based β-ray absorption method (BAM-1020; Met One Instruments Inc.). Volume mixing ratios of SO_2_, CO, O_3_, and NO_2_ were measured with instruments manufactured by Teledyne API using the following technologies: pulsed UV fluorescence (SO_2_), gas filter correlation (CO), UV photometry (O_3_), and chemiluminescence (NO_2_).

### Statistical Modeling

Principal components analysis (PCA), orthogonal partial least-squares discriminant analysis (OPLS-DA), and *T*-distributed stochastic neighbor embedding (*t*-sne) analysis were carried out following the protocol described in the Supporting Information (p. S3).

### Human Nasal Cell Viability Assay

The assay used to assess the viability of human primary nasal epithelial cells exposed to PM_2.5_ extracts is described in detail in the Supporting Information (p. S2).

## Results and Discussion

The back-trajectories and measured ambient parameters (temperature, PM_2.5_ mass, and ambient gas concentrations) over the entire sampling period are summarized in Figures 1 and S1 and discussed in the Supporting Information (p. S4). Briefly, the air–mass back-trajectories and their seasonal frequencies (Figure 1A,B) are similar to previously reported observations at the Gosan Climate Observatory,^38^ and the PM_2.5_ concentrations (Figure 1C) are lower than what Kim et al. observed on Jeju Island between 2015 and 2019,^39^ most likely due to the regional COVID-19 lockdown during the current study’s sampling period.

### Cytotoxicity and Chemical Characterization of Water-Extracted PM_2.5_

For all 85 filters and 30 field blanks, the toxicity of aqueous extracts was examined in primary human nasal epithelial cells (triplicate, 48 h) using fivefold dilutions of the extracts in the nasal cell culture medium. The median cytotoxicity for 30 field blanks was low (0.036%) but ranged up to 10.5% in one batch. To be consistent and conservative, we therefore subtracted 10.5% cytotoxicity from each sample, resulting in 49% of the sample extracts (*n* = 42) with cytotoxicity above maximum blank (Table S6). Interestingly, four of the five most toxic samples were collected in late fall or winter months (November–December 2020 and November 2019). In preliminary tests, three random PM_2.5_ sample filters were sectioned and extracted separately by three separate protocols, including the current aqueous protocol, as well as one with hexane and another with methanol/toluene. Importantly, only the aqueous extracts produced measurable cytotoxicity in the current bioassay (Figure S2); thus, all further characterizations focused on the aqueous extracts. According to the literature, PM_2.5_ extracted using both water-based and polar solvent-based methods showed toxic effects (on cellular and acellular assays).^40−42^ In our samples, the higher cytotoxicity observed with our endpoint (cytotoxicity to human nasal cells) could be attributed to the extraction of chemicals that are more likely to cross biological barriers in human cells.^4,22^

**Figure 2:**
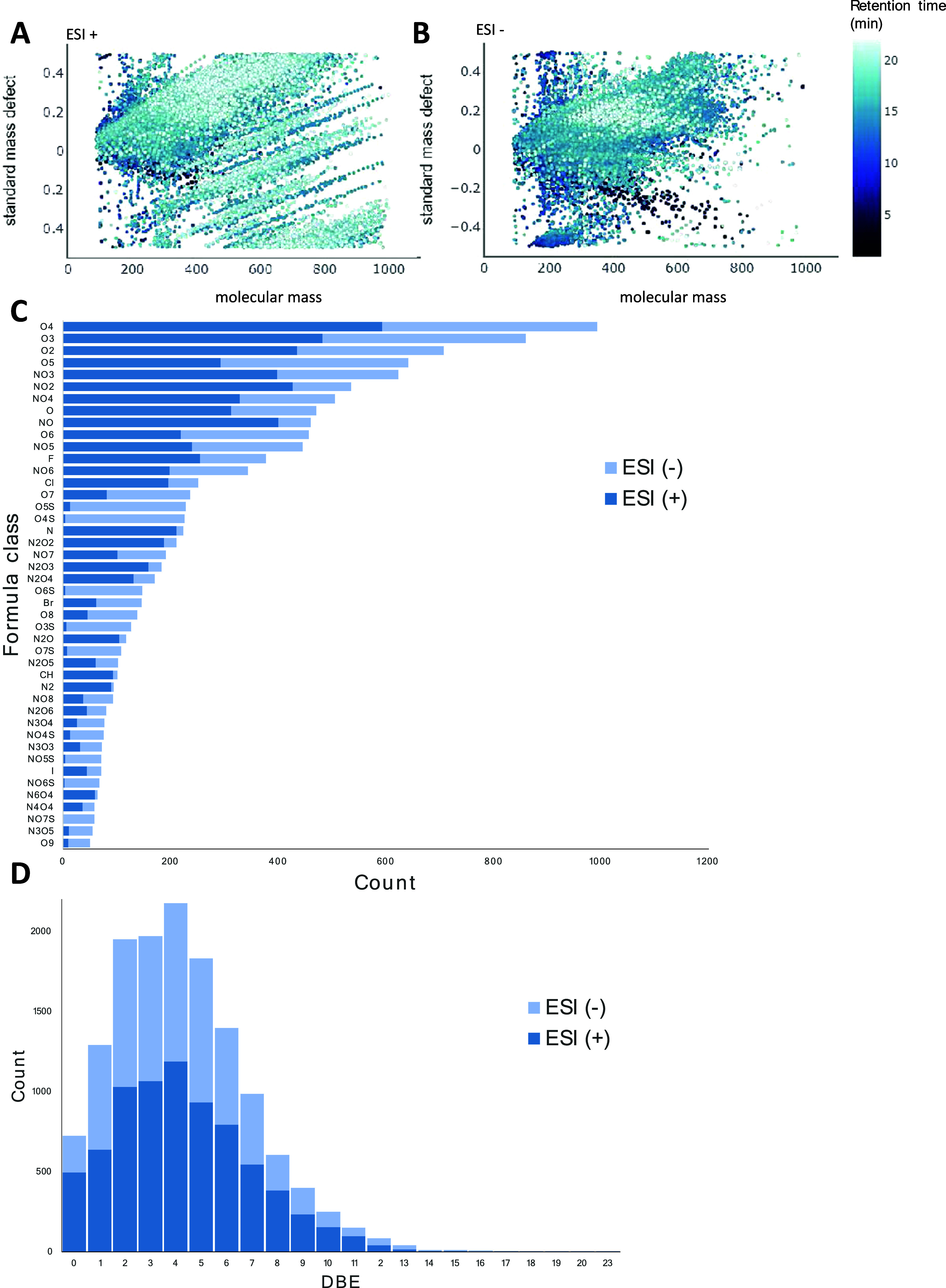
Standard mass defect plots of all nontarget molecular features detected in (A) positive ionization (*n* = 27,724) and (B) negative ionization (*n* = 24,361) modes, colored by retention time. For the combined data sets, the corresponding total number of features with an assigned formula is shown by (C) heteroatomic formula class and (D) number of double bond equivalents (DBEs). Note that the four halogenated formula classes (shown as F, Cl, I, or Br) contain other heteroatoms and multiple halogens, but for simplicity, these are grouped.

**Figure 3:**
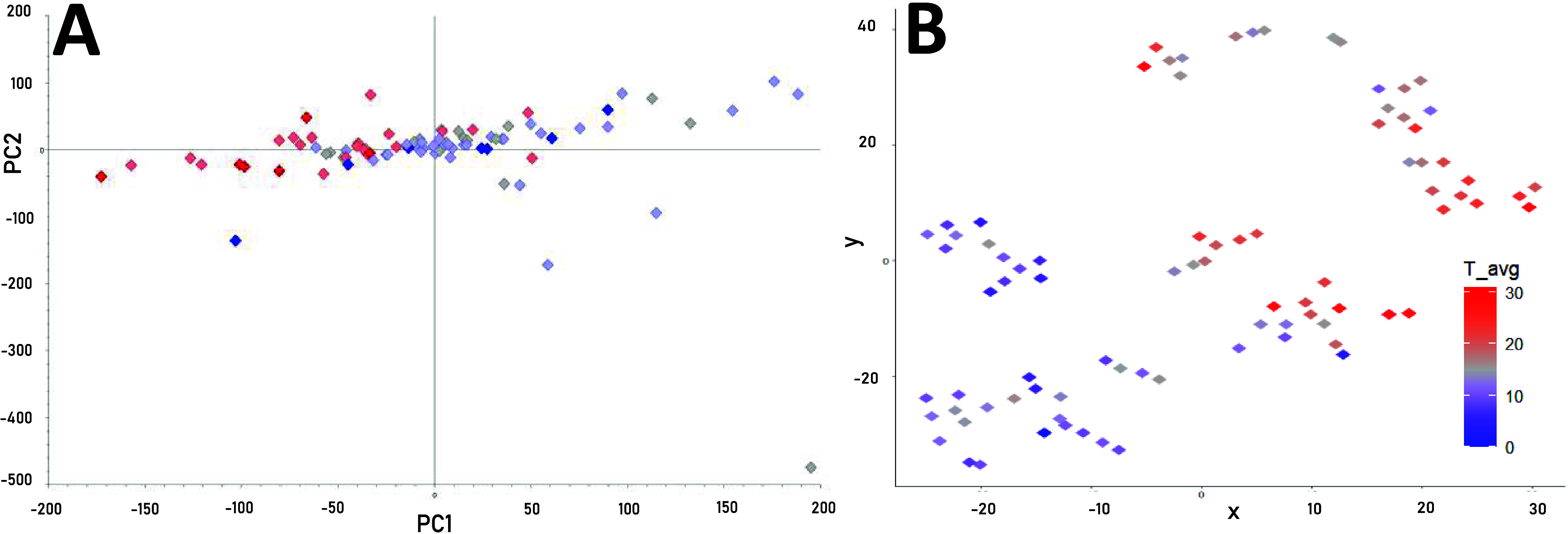
Unsupervised multivariate analyses of the 85 samples using all chemical signals as variables, first by (A) principal component analysis (PCA; PC1 = 0.096 and PC2 = 0.083, *Q*^2^ = 0.0096) and then by (B) *T*-distributed stochastic neighbor embedding (*t*-sne; perplexity = 2 and 2000 steps). Samples are colored by the average temperature at the observatory during collection, with red indicating warmer days and blue indicating cooler days.

The composition of the inorganic components in the water-extracted samples is presented in Table 1, and a detailed discussion is reported in Supporting Results (p. S5). Further characterization of organic compounds in the aqueous extracts by nontarget analysis revealed 52,085 molecular features (Table S5). The overall complexity can be visualized in plots of standard mass defect versus molecular mass, including 27,724 molecular features in positive ionization mode (Figure 2A) and 24,361 in negative ionization mode (Figure 2B). Among all features, 1034 features were redundant (i.e., ionized in both modes) based on the criteria of sharing a common precursor ion mass (±0.001 Da, after accounting for protonation/deprotonation) and retention time (±0.15 min), resulting in a final total of 51,051 unique molecular features. Previous studies have described similarly complex mixtures of WSOCs in PM_2.5_ from south and east Asia.^13,14^ Notably, Papazian et al. also compared the chemical composition of PM_2.5_ extracted by water, polar solvents, and nonpolar solvents and found that the water-extractable substances were the most complex mixture.^14^ Going beyond traditional spectral library screening of detected features, the next sections describe the nontarget analysis workflows uniquely developed for the study, and the statistics applied with all the chemical signals detected (Figure 3).

**Table 1:** Monitored PM and Gas Concentrations at the Observatory during Each Collection Period (*n* = 85) and the Corresponding Detection Frequencies and Concentrations of Detected Metals and Major Ions in Aqueous Extracts of PM_2.5_

analyte	detection frequency (%)	units	median concentration	minimum concentration	maximum concentration
hourly monitored PM and gas concentrations at the observatory over the campaign period					
PM_2.5_	100	μg/m^3^	15.8	6.9	39.2
PM_10_	100	μg/m^3^	31.8	14.8	72.7
SO_2_	100	ppm	0.00074	0.00003	0.00017
CO	100	ppm	0.18	0.08	0.33
O_3_	100	ppm	0.044	0.011	0.064
NO_2_	100	ppm	0.0043	0.0016	0.0089
metals and metalloids analyzed in aqueous extracts of PM_2.5_ (ICP-MS)					
^11^B	100	ng/m^3^	3.88	0.2	24.7
^27^Al	100	ng/m^3^	16.0	0.4	166
^48^Ti	100	ng/m^3^	13.5	2.8	1110
^52^Cr	57	ng/m^3^	0.33	<LOD^a^	101
^55^Mn	100	ng/m^3^	5.68	0.2	23
^57^Fe	100	ng/m^3^	25.1	0.8	1060
^59^Co	14	ng/m^3^	0.03	<LOD	1.3
^60^Ni	63	ng/m^3^	0.50	<LOD	97.2
^63^Cu	94	ng/m^3^	0.97	<LOD	5.6
^66^Zn	100	ng/m^3^	25	1.1	160
^71^Ga	45	ng/m^3^	<LOD	<LOD	0.7
^88^Sr	87	ng/m^3^	0.73	<LOD	53.4
^107^Ag	0	ng/m^3^	<LOD	<LOD	<LOD
^111^Cd	82	ng/m^3^	0.70	<LOD	13.6
^137^Ba	100	ng/m^3^	2.19	0.1	23.8
^205^Tl	82	ng/m^3^	0.21	<LOD	12.4
^208^Pb	100	ng/m^3^	5.20	0.2	21.1
major ions analyzed in aqueous extracts of PM_2.5_ (ion chromatography)					
Cl^–^	100	ng/m^3^	87.1	0.8	3880
NO_2_^–^	62	ng/m^3^	0.77	<LOD	5.1
Br^–^	62	ng/m^3^	3.59	<LOD	4020
NO_3_^–^	78	ng/m^3^	155	<LOD	8550
PO_4_^3–^	79	ng/m^3^	7.09	<LOD	45.3
SO_4_^2–^	71	ng/m^3^	2863	<LOD	5840
Na^+^	100	ng/m^3^	292	43.9	3420
NH_4_^+^	100	ng/m^3^	138	10.8	385
K^+^	100	ng/m^3^	95.2	16.5	363
Mg^+^	0.1	ng/m^3^	0.99	<LOD	2
Ca^+^	0.1	ng/m^3^	1.48	<LOD	2.9

a LOD = limit of detection.

### Nontarget Analysis of Water-Extracted PM_2.5_

Spectral library searches in MassBank Europe and MoNA resulted in 2637 preliminary spectral matches (similarity score >70%, Δ*m*/*z* < 5 ppm). From this list of structural annotations, 39 authentic standards were obtained and analyzed by the current analytical method. From the 39 authentic standards, 16 molecular features were confirmed at level 1 confidence (Table 2), considering the retention index (Figure S4), MS2 spectrum, precursor mass, and isotope abundance. As suggested by Alygizakis et al.,^43^ details of the molecular confirmations are shown (Figures S5–S20), and these included plastic additives (e.g., dibutyl adipate, pentadecanoic acid), personal care products (e.g., *N*-dodecanoyl-*N*-methylglycine, 3,4-dimethoxybenzaldehyde), and pharmaceuticals (e.g., azelaic acid). Nonconfirmed molecular candidates retained only their assigned molecular formula (level 4) but not the structural annotation.

**Table 2:** Summary of the 16 Compounds Confirmed by Standard Injection (Confidence Level 1) and 27 Other Structurally Annotated Features (Confidence Level 2)^a^

ID level	compound name	CAS #	molecular formula	Δ*m*/*z* (ppm)	ΔRTI	ionization mode	feature #
1	orcinol	504-15-4	C7H8O2	0.65	24.53	ESI (−)	450
1	salicylic acid	69-72-7	C7H6O3	0.36	7.63	ESI (−)	758
1	azelaic acid	123-99-9	C9H16O4	0.43	21.68	ESI (−)	2350
1	1-naphthalenesulfonic acid	85-47-2	C10H8O3S	0.87	21.44	ESI (−)	2601
1	3-phenoxybenzoic acid	3739-38-6	C13H10O3	4.18	24.39	ESI (−)	3709
1	pentadecanoic acid	1002-84-2	C15H30O2	0.95	11.93	ESI (−)	5383
1	salazinic acid	521-39-1	C18H12O10	1.42	17.88	ESI (−)	14,887
1	perfluorooctanoic acid	335-67-1	C8HF15O2	0.31	10.85	ESI (−)	16,301
1	perfluorononanoic acid	375-95-1	C9HF17O2	0.73	8.72	ESI (−)	18,543
1	α-pinene	7785-70-8	C10H16	0.44	25.07	ESI (+)	1058
1	(−)-nicotine	54-11-5	C10H14N2	0.37	27.63	ESI (+)	2179
1	3,4-dimethoxybenzaldehyde	120-14-9	C9H10O3	0.12	21.76	ESI (+)	2391
1	dehydrocostus lactone	477-43-0	C15H18O2	1.69	5.97	ESI (+)	6033
1	bentazone	25057-89-0	C10H12N2O3S	2.45	1.56	ESI (+)	6651
1	dibutyl adipate	105-99-7	C14H26O4	2.01	8.93	ESI (+)	7824
1	anthranilic acid	118-92-3	C7H7NO2	3.33	26.59	ESI (+)	1295
2	phthalic anhydride	85-44-9	C8H4O3	0.81	19.35	ESI (+)	1784
2	isophorone	78-59-1	C9H14O	1.37	12.42	ESI (+)	1364
2	tryptamine	61-54-1	C10H12N2	1.68	10.93	ESI (+)	2315
2	aspirin	50-78-2	C9H8O4	0.61	0.77	ESI (+)	3456
2	dicyclohexylamine	101-83-7	C12H23N	0.16	1.08	ESI (+)	3060
2	syringaldehyde	134-96-3	C9H10O4	1.04	29.14	ESI (+)	3597
2	3,5-di-*tert*-butyl-4-hydroxybenzaldehyde	1620-98-0	C15H22O2	0.30	6.96	ESI (+)	7372
2	di-*n*-butyl phthalate	84-74-2	C16H22O4	0.64	22.01	ESI (+)	10,746
2	tebupirimfos	96182-53-5	C13H23N2O3PS	1.82	6.46	ESI (+)	11,915
2	tri-*n*-butyl acetyl citrate	77-90-7	C20H34O8	0.40	26.36	ESI (+)	18,923
2	2-phenylglycine	2835-06-05	C8H9NO2	0.15	3.64	ESI (+)	1043
2	γ-dodecalactone	2305-05-07	C12H22O2	2.06	4.18	ESI (+)	4003
2	6-methylcoumarin	92-48-8	C10H8O2	1.61	8.2	ESI (+)	2053
2	4-methylphthalic anhydride	19438-61-0	C9H6O3	0.55	28.58	ESI (+)	2143
2	cinnamamide	621-79-4	C9H9NO	0.61	27.69	ESI (+)	1519
2	phthalic anhydride	85-44-9	C8H4O3	0.81	19.35	ESI (+)	1551
2	4-hydroxycoumarin	1076-38-6	C9H6O3	0.18	1.51	ESI (+)	2142
2	coumarin	91-64-5	C9H6O2	1.43	18.83	ESI (+)	1474
2	succinic acid	110-15-6	C4H6O4	4.36	26.22	ESI (−)	348
2	4-nitrocatechol	3316-09-04	C6H5NO4	0.45	24.40	ESI (−)	1191
2	*p*-toluenesulfonic acid	104-15-4	C7H8O3S	1.75	21.98	ESI (−)	1770
2	suberic acid	505-48-6	C8H14O4	0.35	18.66	ESI (−)	1867
2	di-*n*-butyl phthalate	84-74-2	C16H22O4	0.32	18.98	ESI (−)	8056
2	3-hydroxybenzoic acid	1999-06-09	C7H6O3	2.48	10.61	ESI (−)	738
2	benzoic acid	65-85-0	C7H6O2	1.16	11.91	ESI (−)	406
2	phenylparaben	17696-62-7	C13H10O3	2.11	14.82	ESI (−)	3709
2	4-nitrocatechol	3316-09-04	C6H5NO4	0.45	24.4	ESI (−)	1123

a ΔRTI = difference between feature and reference standard’s RTI. Δ*m*/*z* = difference between feature and reference standard’s precursor mass.

**Figure 4:**
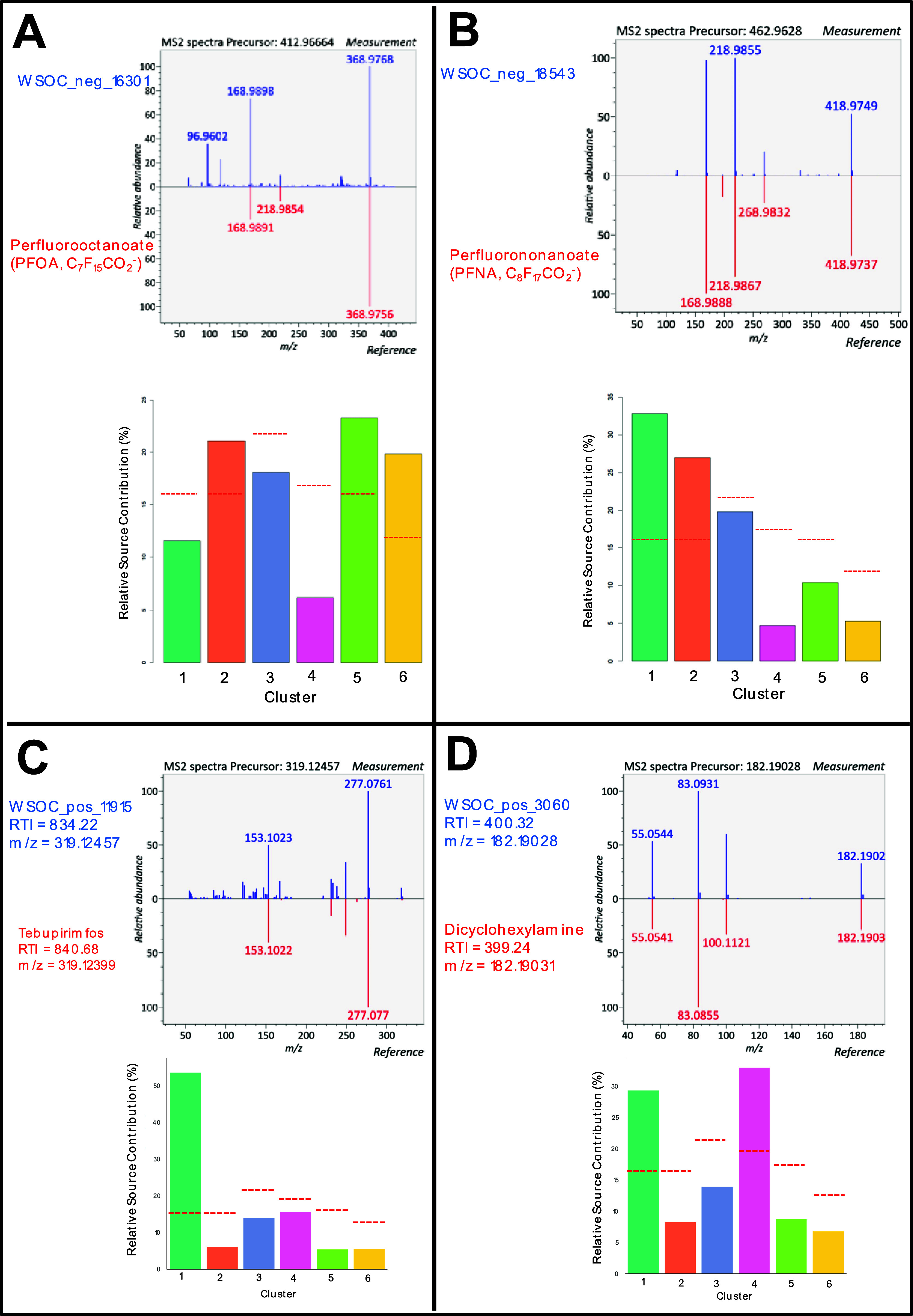
Example MS2 spectral library matches (top of each panel) of four nontarget features compared to the reference spectra of PFOA (A), PFNA (B), tebupirimfos (C), and dicyclohexylamine (D). Both PFOA (A) and PFNA (B) were confirmed with authentic standards, level 1 confidence. The relative source contribution of each back-trajectory cluster region for each substance (bottom of each panel) is also shown, estimated by multiplying peak areas with the relative contribution of air coming from each source region in each sample. The red dashed lines show the relative frequency (%) that air masses originate from each back-trajectory region; thus, source contributions above or below these red lines indicate regions whose air is relatively enriched or depleted in the substance(s), respectively. The six back-trajectory clusters represent different source regions for the air reaching the sampling station: 1—East Sea/Sea of Japan; 2—East China Sea; 3—Korean Peninsula; 4—Shandong/Beijing; 5—North China; 6—North China/Mongolia.

To estimate the geographic sources of any feature, the normalized peak area can be multiplied by the proportional weight of each back-trajectory source region for each sample (Figure 1B), and the results are summed over the campaign. Example results are shown in Figure 4, including for two level 1 confirmed substances, perfluorooctanoic acid (PFOA, Figure 4A) and perfluorononanoic acid (PFNA, Figure 4B). The results suggest that the primary geographic source regions for PFOA are northern China (clusters 5 and 6), which together account for 43% of the PFOA signal; this is notable as these regions’ combined frequency in back-trajectories was only 28% (i.e., source contributions from regions 5 and 6 both exceed the red lines in Figure 4A). In contrast, the primary source region for PFNA was the East Sea/Sea of Japan (region 1), which accounted for 33% of the PFNA signal, even though air coming from this region contributed only 16% of the time overall. These example results are consistent with known manufacturing sources of these two substances in the region: specifically, China became the primary global manufacturing site for PFOA after 2002, and Japan was a major historical producer of PFNA.^44^ Due to their current levels in environmental samples, extreme environmental persistence, and mobility in the atmosphere, it has been argued that these two PFASs are planetary boundary threats (e.g., ref (45)), yet the environmental occurrence of most PFAS has not been monitored and nontarget screening for PFAS may help to prioritize future targeted studies.

An additional 28 features were annotated (level 2 confidence) based on the combination of high-quality MS2 spectral library match (score >70%), precursor mass in MS1 (Δ*m*/*z* < 5 ppm), and retention index (ΔRTI < 30) (Table 2 and Figures S22–S25). RTIs of library matches were retrieved from the online Norman Substance Database, and RTIs of the features were calculated using the RTI calibration solution analyzed with the samples (Figure S4). Notable level 2 annotated features included insecticide tebupirimfos (Figure 4C), which has not previously been reported in PM_2.5_ to our knowledge, and dicyclohexylamine (Figure 4D), a tire-derived substance^46^ that was previously detected in aqueous extracts of PM_2.5_ in South Asia.^14^ Both compounds had intense signals in days impacted by region 1 (East Sea/Sea of Japan), and dicyclohexylamine was also more prominent in days impacted by region 4 (Shandong/Beijing). Aspirin (acetylsalicylic acid) was also among the level 2 annotations and interestingly is known to be transformed to one of the confirmed (i.e., level 1) compounds, salicylic acid. Although salicylic acid can be naturally produced by plants, seeing its signal correlated to the signal of aspirin increases confidence in both annotations and suggests that the sources of both substances detected here were largely anthropogenic. Moreover, looking at the normalized peak areas from these two compounds throughout the current campaign, their quantitative responses were moderately correlated (Pearson’s *R* = 0.5, *p* = 9.6 × 10^–7^, Figure S21).

For molecular formula assignment of unknown features, we developed a completely new workflow: 50 reference standards (Table S2) were first used to validate the performance of two software tools (SIRIUS and MS-FINDER) with the current data set. These included 21 standards injected in positive ionization mode and 29 in negative ionization mode, all having a wide range of molecular mass (121–916 Da), chemical functionalities, and molecular formulas including various heteroatoms (P, S, N, and O) and halogens (F, Cl, Br, and I). Considering the top two ranked predicted molecular formulas for each reference standard, both SIRIUS and MS-FINDER performed well, with accurate formulas predicted by both tools as the top-ranked for 76% (38 of 50) of substances (Table S2). Moreover, for all 50 reference standards, the accurate formula was predicted by at least one of the tools, either as top-ranked or second-ranked. Performance was even better considering the subset of substances that did not contain a F atom, in which case the accurate formula was predicted by both tools as the top-ranked for 88% (38 of 43) of reference standards. Given the possibility that other fluorinated substances may be missed by this workflow, a secondary workflow was applied to identify highly fluorinated substances.

Based on excellent performance for most reference standards, we assigned a molecular formula to unknown features when both SIRIUS and MS-FINDER predicted the same molecular formula, ranked as the top 1 or 2. Among all detected molecular features, 13,907 (27%) were thereby assigned a molecular formula (level 4 confidence^36^) by these conservative criteria. Over the whole campaign, the most commonly detected molecules in the aqueous extracts of PM_2.5_ belonged to simple oxygenated formula classes (i.e., C_*x*_H_*y*_O_Z_), dominated by O_4_ > O_3_ > O_2_ > O_5_ in both positive and negative modes (Figure 2C). Nevertheless, more than half of the assigned formulas (55%) contained at least one nitrogen atom, including prominent classes with mixed nitrogen and oxygen (e.g., C_*x*_H_*y*_NO_*z*_ and C_*x*_H_*y*_N_2_O_*z*_, where *z* = 1–6). A range of mixed sulfur- and oxygen-containing molecules, detected almost exclusively in negative mode (i.e., SO_*x*_^–^, where *x* = 3–7, Figure 2C), were indicative of organic sulfonates and sulfates. Papazian et al. previously categorized N- and S-containing molecules as among the molecular hallmarks of polluted air,^14^ and in PM_2.5_ collected in Beijing, Ma et al. reported seasonal trends with higher abundance of organosulfates in summer and more abundant nitrogenated compounds in winter.^15^ It is only notable here that the current campaign was biased toward winter months (43 of 85 samples), as the 16 month period covered two winters (Figure 1B). The number of double bond equivalents (DBEs) in annotated molecules was also calculated from each assigned formula (i.e., DBE = C + 1 – (H/2) – (*X*/2) + (N/2); *X* = number of halogens). The distribution of DBEs in both modes suggested a preponderance of molecules with at least four DBEs (representing 57% of all formulas), which may therefore contain aromatic structures but also a large number of nonaromatic substances (DBEs < 4, representing 43% of all assigned formulas) and, to a lesser extent, aliphatic structures (DBE = 0, 4% of formulas) (Figure 2D). The assigned formulas for hundreds of substances also contained F (*n* = 357, including the flagged PFAS described later) or Cl (*n* = 251) (Figure 2C), notably overcoming a limitation for halogens in previously described workflows for molecular formula assignments in PM_2.5_.^14,15^

**Figure 5:**
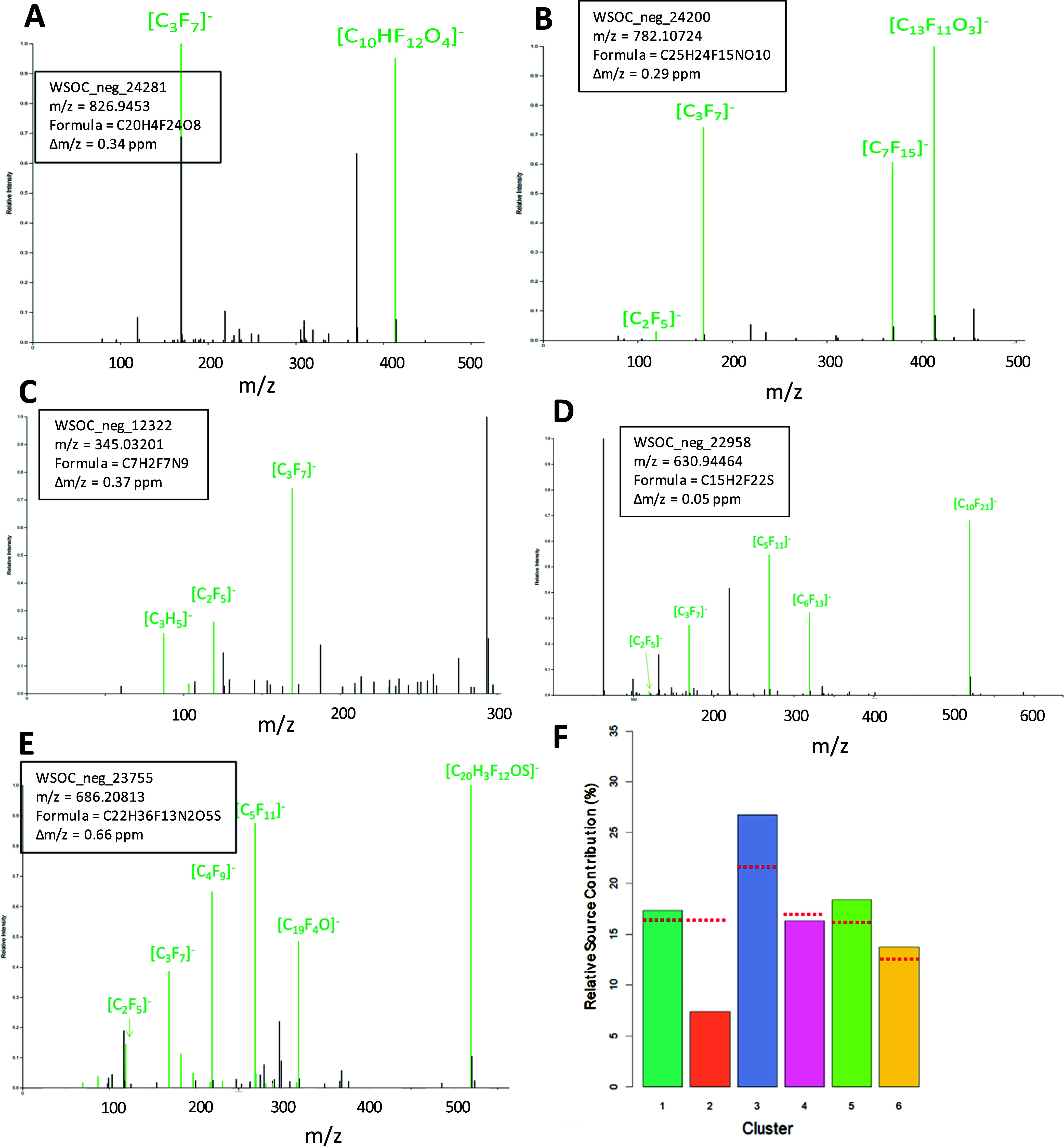
Deconvoluted MS2 spectra (A–E) of five example PFASs (out of 74 total) discovered in aqueous extracts of PM_2.5_ by the workflow incorporating SIRIUS formula prediction and manual MS2 fragment searching. Green spectral fragments are those used by SIRIUS to assign the molecular formula. (F) Considering all unknown PFAS features together, the major source region was the Korean Peninsula (cluster 3), and the least important was the cleanest air coming from the China Sea (cluster 2). The red dashed lines show the relative frequency (%) that air masses originate from each back-trajectory region; thus, source contributions above or below these red lines indicate regions whose air is relatively enriched or depleted in the substance(s), respectively. The six back-trajectory clusters represent different source regions of the air reaching the sampling station: 1—East Sea/Sea of Japan; 2—East China Sea; 3—Korean Peninsula; 4—Shandong/Beijing; 5—North China; 6—North China/Mongolia.

This general workflow used here for molecular formula assignments was shown to underestimate the number of fluorinated compounds. This was because the workflow relied on a consensus between MS-FINDER and SIRIUS, but MS-FINDER performed poorly for all fluorinated reference standards. Nevertheless, SIRIUS accurately predicted the molecular formulas (top-ranked) of all six PFAS reference standards in validation testing (i.e., C_6_HF_11_O_2_, C_7_HF_13_O_2_, C_8_HF_15_O_2_, C_9_HF_17_O_2_, C_4_HF_9_O_3_S, and C_8_H_2_F_17_NO_2_S; Table S2). Thus, a separate workflow using SIRIUS and MS-DIAL was developed here. Among all unknown features in the current study, 6122 were initially predicted to contain at least two fluorine atoms by SIRIUS, thus each plausibly corresponding to a theoretical PFAS (i.e., containing a −CF_2_– moiety). In previous work, PFAS have been successfully identified in water, biota, and particulate matter by manual searches for common perfluoroalkyl fragments.^46−50^ Thus, using the MS/MS fragment searcher tool in MS-DIAL, 13 common PFAS fragments ([C_*x*_F_2*x*+1_]^−^; *x* = 2–14, Table S3) were searched in the MS2 spectra of all unknown features in negative ionization mode (tolerance of 0.01 Da, ion abundance threshold >10%), and 97 features contained at least two common PFAS fragments. Of these, 74 also had a top-ranked molecular formula corresponding to a PFAS, as predicted by SIRIUS (Table S4). Some of these corresponded to spectra of known PFASs (e.g., perfluoroundecanoate, C_10_F_21_CO_2_^–^), which were identified by suspect screening (using spectral libraries), but most corresponded to unknown PFASs with no spectral match (Figure 5). Together with other recent discoveries, this demonstrates the prevalence of unexpected and unknown PFASs in our environment.^51^

Although the exact structures of these highly fluorinated substances remain uncertain, the diagnostic perfluoroalkyl fragments confirm that these are PFASs. Moreover, given that the structures of these unknown PFASs contain long perfluoroalkyl fragments, all of these substances are likely environmental precursors to persistent perfluorinated acids. For example, like perfluoroundecanoate, the spectrum of the unknown PFAS in Figure 5D contains a C_10_F_21_ moiety. By analogy to the atmospheric oxidation of 10:2 fluorotelomer alcohol (C_10_F_21_CH_2_CH_2_OH), this unknown substance could plausibly yield perfluoroundecanoate in the atmosphere through hydroxyl radical oxidation processes.^52^ Similarly, the example substances shown are likely precursors to PFOA (Figure 5B), perfluoropropionate (Figure 5C), and perfluoropentanoate (Figure 5E) in the environment.

In general, the source region for these 74 prospective PFAS was primarily the Korean Peninsula (region 3), followed by China (regions 4–6), with the least coming from the East China Sea (region 2) (Figure 5F). Figure 5F also shows a smaller contribution of PFASs from air masses originating from the Sea of Japan (region 1). This could be due to the documented production of PFASs in Japan^53^ and emissions via sea spray aerosols.^54^ Overall, these data suggest that the sources of these unknown PFASs may be widespread and possibly linked to their contemporary use in several areas of East Asia.

### Effects of Atmospheric Conditions and Origin of the Air on Chemistry

To broadly examine for relationships between chemistry, back-trajectories, and ambient temperature, all chemistry data (inorganic and all WSOC nontarget features) were combined in unsupervised multivariate models. An initial PCA analysis showed no strong grouping of the samples (Figure 3A), with PC1 and PC2 each explaining less than 10% of the overall variability, and *Q*^2^ was lower than 0.01. However, by coloring the samples by average temperature during collection, it was evident that PC1 (9.6% of variation) partially separated the samples, with samples collected on warmer days grouping negative on PC1, while those from cooler days grouping positive on PC1. The potential influence of each back-trajectory source region on the detected chemistry was similarly investigated, but no separation of the samples was found (data not shown). In a follow-up *t*-sne analysis—an alternative unsupervised model with the potential to detect groupings based on smaller variations in the data set^55^—more distinctive grouping of the samples was achieved; again, the grouping was to some extent correlated with average temperature (Figure 3B), indicating that the grouping observed with the *t*-sne analysis is due to a subset of variables among all of the chemical signals detected. The same *t*-sne output was replotted by sizing each sample marker by the relative contribution of the two maritime regions (Figure S3A), the Korean Peninsula (Figure S3B), Shandong/Beijing (Figure S3C), or the two North China source regions (Figure S3D). These combined results suggested that variations in the complex chemistry could be attributed to the combination of warmer temperatures and maritime source regions in the summer (Figure S3A) and cooler temperatures and northern China source regions in the winter (Figure S3D).

It was not possible in this study to disentangle the independent influences of temperature and back-trajectory factors on sample chemistry because both factors are correlated and influenced by season (e.g., in summer, the air is warmer and mainly comes from maritime regions, while in winter, the air is cooler and often comes from China, Figure 1). Nevertheless, in the next sections, we applied supervised multivariate models to discriminate which chemicals are most influenced by warm versus cool temperatures or maritime versus continental back-trajectories.

**Figure 6:**
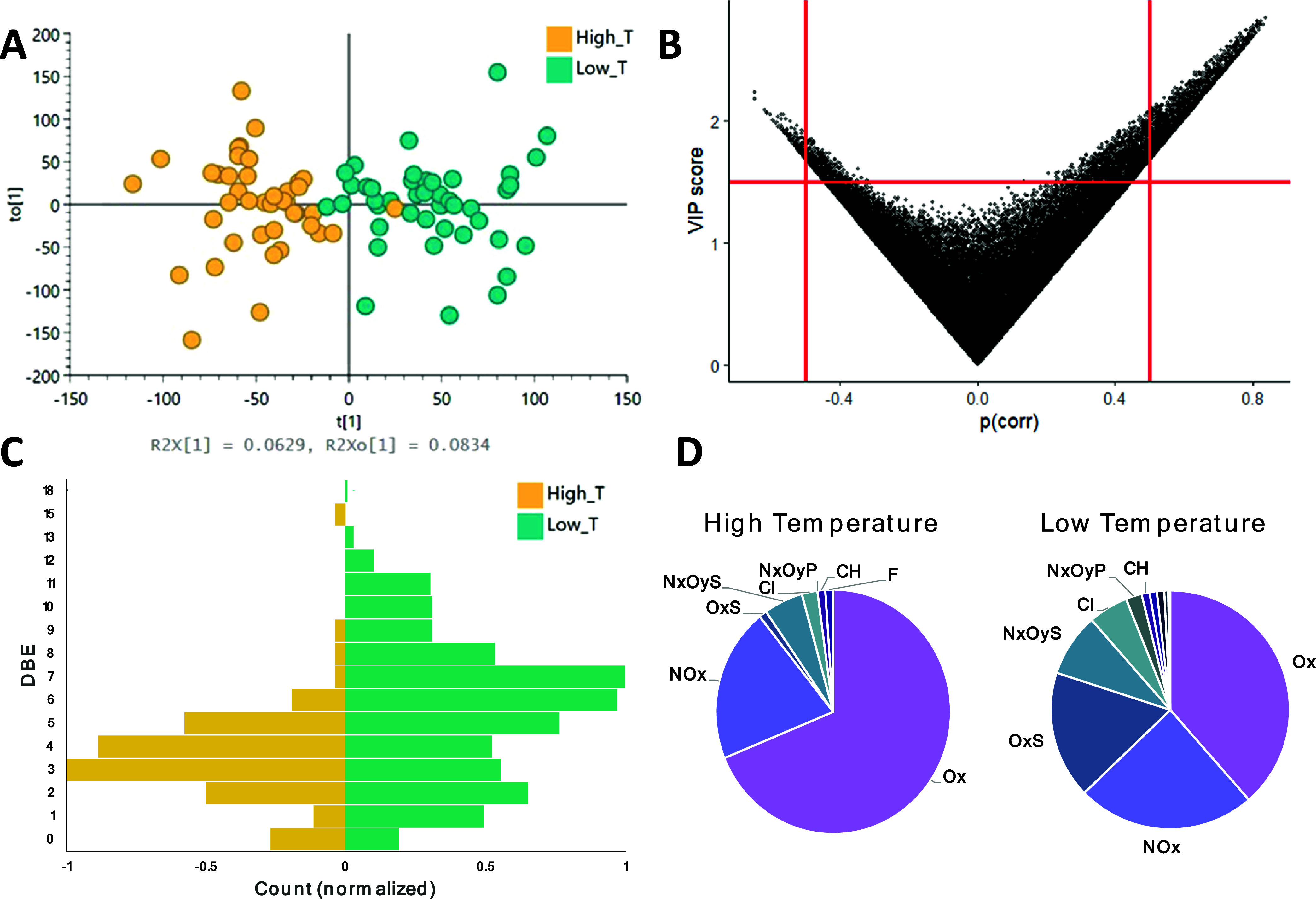
(A, B) Output of an OPLS-DA model contrasting all of the chemical signals (52,113 variables) measured in PM_2.5_ extracts (*n* = 85) taken on high average temperature days (yellow) and low average temperature days (teal), including (A) score plot showing separation of the samples (*R*^2^ = 0.733 and *Q*^2^ = 0.472) and (B) scatter plot of the variable’s influence on projection (VIP) score versus correlation coefficients, pCorr, for all nontarget features. The subset of features with VIP score >1.5 and |pCorr| > 0.5 are highlighted, corresponding to features most correlated with warmer (high *T*; pCorr >0.5) or colder (low *T*; pCorr <0.5) temperatures. Molecular characterization of high-*T* and low-*T* substances are contrasted by distributions of (C) double bond equivalents (DBEs) in bar charts and (D) assigned molecular formula classes in pie charts.

To determine which organic or inorganic substances (*X* variables) were most associated with temperature, an OPLS-DA model was built, with each sample classified according to temperature (*Y* variable). The average temperature across the campaign was 15.0 °C; thus, samples were dichotomized as either higher temperature (average temperature during collection >15 °C, *n* = 42) or lower temperature (average temperature <15 °C, *n* = 45). The OPLS-DA model output generally separated samples by temperature (Figure 6A), with a good fit to the data (*R*^2^ = 0.733) and acceptable robustness (*Q*^2^ = 0.472). Kim et al. described a change in the optical properties of PM_2.5_ at the current site in winter (with the highest aerosol scattering and second-highest aerosol absorption attributed to winter months), suggesting a different chemical composition of aerosols in winter compared to other months.^24^ Interestingly, one particular sample from higher temperature collection days was grouped with samples from lower temperature days, but this unique sample was taken during an unusually warm period in winter (average temperature 16.0 °C, December 6–8, 2020), suggesting that solar intensity (together with temperature) could be an underlying factor in the chemistry of PM_2.5_. Photochemical oxidation of water-soluble brown carbon was recently demonstrated during atmospheric transport of PM_2.5_ from the Indian subcontinent to an Indian Ocean receptor site.^37^

Among all chemical variables, none of the metals or major ions were highly correlated with temperature. However, 2221 nontarget features were highly correlated with a lower temperature (11 annotated at level 2), while only 288 features (1 annotated at level 2) were highly correlated with a higher temperature. Molecular formulas were confidently assigned to approximately half of the highly correlated features; specifically, 53% of features correlated with lower temperatures, while 47% of features correlated with higher temperatures. Among this subset of molecules, those correlating with lower temperatures had significantly higher DBEs (mean DBE = 5.5) than those correlating with higher-temperature days (mean DBE = 3.6; *p* = 0.000011, Figure 6C). To some extent, these differences may relate to seasonal shifts in back-trajectory sources of the air because more polluted continental air arrives at the observatory on cooler days than on warmer days. Nevertheless, this may also indicate that more aromatic and cyclic structures are present on colder days when photooxidation potential is lower, consistent with the fact that highly oxygenated compounds (i.e., O_6_–O_9_) were highly correlated with warmer temperatures (Figure 6D). As a simple analogy, the photooxidation of benzene (DBE = 4) is initiated by hydroxyl radical and proceeds through ring-opening to form oxidized molecules with lower DBEs, e.g., butanediol (DBE = 3, O_2_), 2,3-epoxy-butandiol (DBE = 3, O_3_), and glyoxal (DBE = 1, O_2_).^56^ To summarize, it is likely that seasonal changes in photooxidation and back-trajectory source regions together contribute to the observed differences in PM_2.5_ chemistry on cooler and warmer days.

The *t*-sne analysis also suggested that the chemistry of the aqueous PM_2.5_ extracts was different when influenced by the two maritime back-trajectory source regions (Figure S3A). Thus, in a separate OPLS-DA model (Figure 6) using the same chemical *X* variables, we dichotomized the samples according to whether they were predominantly influenced by the combination of maritime back-trajectory regions (*n* = 21 samples) or the four continental regions (*n* = 64). The resulting OPLS-DA model was robust (*Q*^2^ = 0.37) and explained a high proportion of the variation (*R*^2^ = 0.721). Of the 2157 variables strongly correlating with maritime source regions, all were nontarget features, including one identified at level 1 by reference standard injection (3,4-dimethoxybenzaldehyde) and two others annotated at level 2 (MS2 similarity >70%, Δ*m*/*z* < 5 ppm, and ΔRTI < 30), specifically 2-phenylglycine and acetylsalicylic acid (i.e., aspirin). Aspirin (pharmaceutical) and 3,4-dimethoxybenzaldehyde (used in personal care products) have anthropogenic sources and are likely released through wastewater to the coastal sea environments. Thus, sea spray aerosol generation might explain their presence in PM_2.5_, as described by Johansson et al. for certain PFASs.^57^ Interestingly, 2-phenylglycine was also correlated with high-temperature days, and to our knowledge, this is the first time its presence in ambient air has been reported.

**Figure 7:**
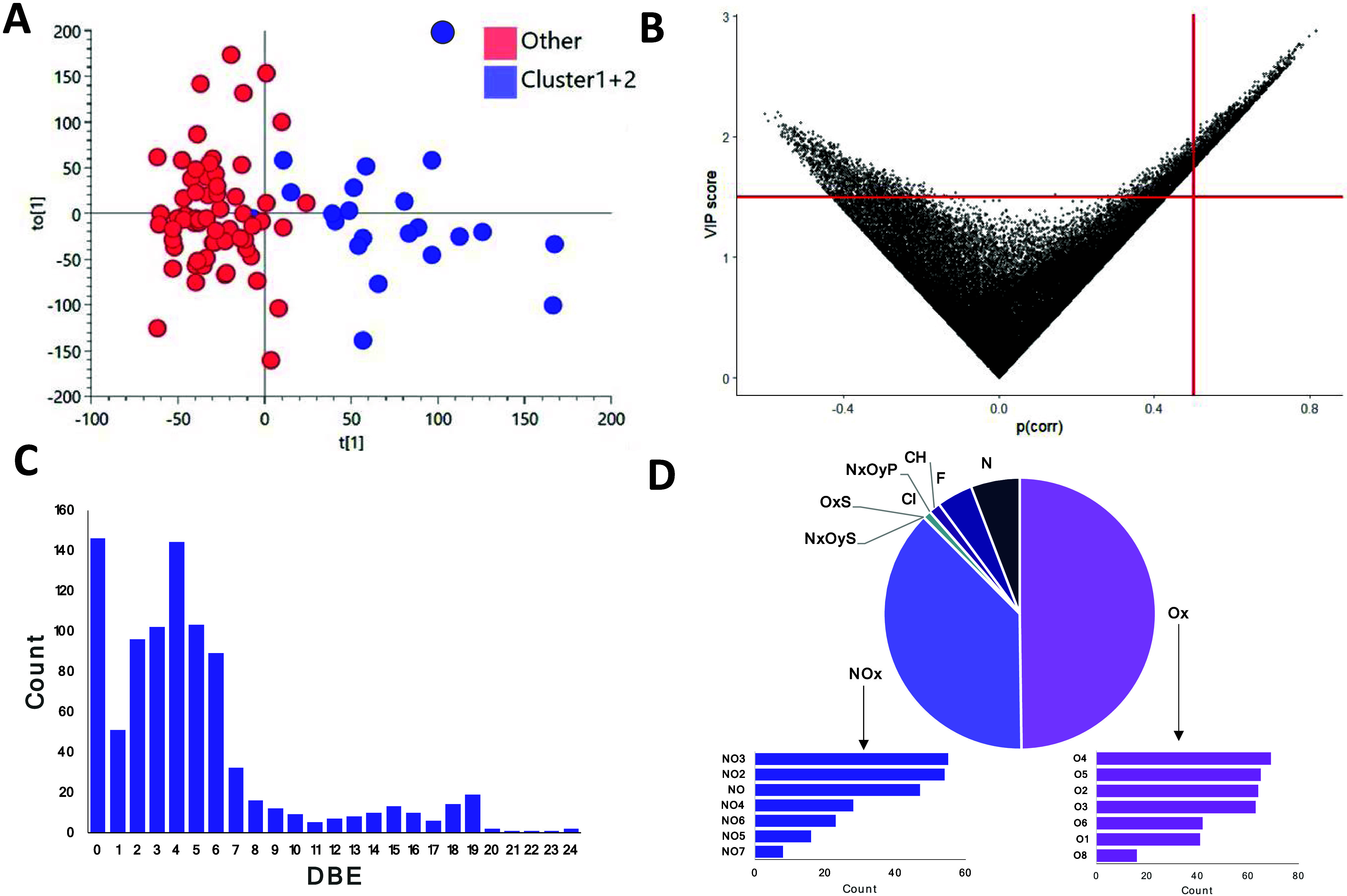
Output of an OPLS-DA model contrasting the WSOC profiles of all PM_2.5_ extracts (*n* = 85) impacted primarily from maritime regions (regions 1 and 2) to those impacted primarily from continental source regions (regions 3–6), including the (A) score plot showing separation of the samples (1 + 1, *R*^2^ = 0.721 and *Q*^2^ = 0.37) and (B) scatter plot of the variable’s influence on projection (VIP) score versus correlation coefficients, pCorr, for all nontarget features. Nontarget features with pCorr >0.5 and VIP > 1.5 are highlighted in (B), corresponding to those molecules most positively correlated with the maritime source regions whose molecular characteristics are shown by distributions of (C) double bond equivalents (DBEs) in a bar chart and (D) molecular formula class distributions.

Among features correlating with the maritime source regions, 55% had a confidently assigned molecular formula (Figure 7C,D). Among these molecules, those correlating with maritime regions were dominated by saturated compounds (i.e., DBE = 0) and lower numbers of DBEs (i.e., 2 < DBE < 6, Figure 7C), which is lower than the overall DBE distribution of nontarget features (Figure 2C) and lower than the DBE distributions for features correlated with both high and low temperatures (Figure 6C). The predominant formula classes among features highly correlated with maritime regions were low to moderately oxygenated compounds (i.e., O_2_–O_5_), followed by nitrogen-containing oxygenated substances (NO–NO_3_, Figure 7D). It has been reported that commercial shipping (i.e., cargo and tanker ships) can emit nitrogenated organic chemicals,^58,59^ and numerous shipping routes pass in the vicinity of Jeju Island.^60^

### Limitations and Future Directions

The present study confirms that the cytotoxic water-soluble fraction of PM_2.5_ has a complex chemical composition that is seasonally influenced by regional meteorology and shifting back-trajectories. This seasonal variability was primarily in the complex profiles of water-soluble organic compounds, and a robust workflow for molecular formula assignment and novel molecular discovery was effectively demonstrated. For research to progress on the molecular drivers of PM_2.5_ toxicity, the current results emphasize that detailed PM_2.5_ chemical characterization should be conducted concurrently with toxicological testing, as studies relying only on total PM_2.5_ concentrations will be blind to the underlying variability and specific chemical differences in their test materials. Considering that half of the current extracts (*n* = 42) were cytotoxic to human nasal cells, further research is needed to identify the key chemical drivers of toxicity in aqueous extracts of PM_2.5_ by applying comprehensive effects-based methods^61,62^ in other relevant human cells (e.g., lung cells) with more detailed mechanistic or molecular-level endpoints (e.g., for oxidative stress).

It is also important to note that the start of the current sampling campaign, in late 2019, approximately coincided with the onset of the COVID-19 pandemic, which spread globally by early 2020. Therefore, the majority of our sampling period was during the pandemic when neighboring countries underwent different extents of lockdown, which likely influenced the current results. As discussed, PM_2.5_ concentrations during the current campaign were lower than what was previously reported at this site, and it is likely that the chemistry of the PM_2.5_ was also influenced by diminished industrial activity and major societal changes in east Asia. Nevertheless, samples were still shown to be toxic to human nasal cells, in particular the complex mixture of substances present in the water-soluble fraction.

## Supporting Information Available

Tables as individual spreadsheets (XLSX)
Text document containing the supporting information and methods, results, and figures (PDF)
