# Supplementary material for: Exploring the Chemical Complexity and Sources of Airborne Fine Particulate Matter in East Asia by Nontarget Analysis and Multivariate Modeling
Source: Environ Sci Technol. 2025 Jan 27;59(5):2623–40. doi: 10.1021/acs.est.4c09615 (PMC11823462; doi:10.1021/acs.est.4c09615)
Supplement: Supplementary file 2 — es4c09615_si_002.pdf [file es4c09615_si_002.pdf]

Exploring the Chemical Complexity and Sources of Airborne Fine Particulate Matter in East Asia by Nontarget Analysis and Multivariate Modeling

Jean Froment[a,b], Jong-Uk Park[c], Sang-Woo Kim[c], Yoonjin Cho[e], Soobin Choi[e], Young Hun Seo[d], Seungyun Baik[d*], Ji Eun Lee[e*], Jonathan W. Martin[a*].

[a] *Department of Environmental Science, Stockholm University, Stockholm, 10691, Sweden*

[b] *NILU, Department of Environmental Chemistry and Health Effects, Kjeller, 2027, Norway*

[c] *School of Earth and Environmental Sciences, Seoul National University, Seoul 08826, Republic of Korea*

[d] *Environmental Safety Group, Korea Institute of Science and Technology (KIST) Europe, Campus E 7.1, Saarbrucken 66123, Germany*

[e] *Chemical & Biological Integrative Research Center, Biomedical Research Division, Korea Institute of Science and Technology, Seoul 02792, Republic of Korea*

*Corresponding authors: Seungyun Baik (sbaik@kist-europe.de), Ji Eun Lee (jelee9137@kist.re.kr), and Jonathan W. Martin (jon.martin@aces.su.se)

Summary: 32 pages, 25 figures, 7 tables (on separate file)

Supplementary Materials:

In this document:

Supplementary methods p.S2-3

Supplementary results p.S4-6

Supplementary figures p.S7-31

Supplementary reference p.S32

Separate file:

Supplementary tables (Tables S1-S7, xlsx).

Supplementary methods

Chemicals. HPLC grade water for extracting filters was from Honeywell AB (Bromma, Sweden). Methanol (Optima grade) and formic acid (purity > 90%) were from VWR. For the internal standards, phenylglyoxylic acid-d5 (purity of 99.78%), daidzein-d6 (purity of 98.00%), N-acetyl-d3-S-(N-methylcarbamoyl)-L-cysteine (purity of 95%), norharmane-d7 (purity of 98.00%), 4-(methylnitrosamino)-1-(3-pyridyl)-1-butanol-d5 (purity of 99.59%), and racemic enterolactone - $^{13}C_3$ (purity of 95.00%) were from Toronto Research Chemicals. Atrazine-2-hydroxy-D3 (purchased from Qmx Laboratories, purity of 99.00%), atrazine-D5 (purchased from Merck, purity of 99.5%) and all the chemicals were stored according to the recommendation from the suppliers upon arrival. For spike-recovery experiments, 13 compounds were acquired and used: monoethyl phthalate (Toronto Research Chemicals, 99.20%), bentazone (Chemtronica AB, 98.10%), rupestonic acid (Ambinter, 98%), tetradecylsulfate (Merck, 95%), phthalic acid (Merck, 98.50%), benzimidazole (Merck, 99.50%), 5,6-dimethylbenzimidazole (Merck, 99.40%), nootkatone (Merck, 99.30%), carbofuran (Santa Cruz Biotechnology, 99.50%), salbutamol (Merck, purity > 96%), dibutyl adipate (Merck, 99.70%), benzoylecgonine (Merck, 99.90%), tris(2-butoxyethyl)-phosphate (Thermo Scientific Chemicals, 98.10%). For biotesting, dimethyl sulfoxide (DMSO) was purchased from Sigma-Aldrich (Burlington, USA, 99.7%).

Other extraction protocols. In preliminary tests, 1/4 of one field blank and three $PM_{2.5}$ samples taken prior to the main campaign were extracted by three separate solvent conditions to identify the fraction with greatest toxicity to human nasal epithelial cells. For water extracts, 1/4 piece of each filter was extracted using 20 mL of HPLC grade water and sonicated at room temperature for 30 min and centrifuged for 10 min at 1800 × g. In addition to the aqueous extracts, following the protocol of Papazian *et al.* [12] we used organic solvents to extract polar organic compounds and non-polar organic compounds by accelerated solvent extractor (ASE, Thermo Fischer, model 350) for 1/4 piece of each filter. The polar compounds were extracted by methanol (10 mL at 150 °C) followed by toluene (10 mL at 150 °C) at 1500 psi, while non-polar organic compounds were extracted using two cycles of 10 mL of Hexane at 100 °C and 1500 psi each cycle. The solvent extracts were filtered with 0.2 µm cellulose syringe filters and evaporated to dryness under a gentle stream of nitrogen at room temperature, and frozen until toxicity testing. For the human nasal cell cytotoxicity testing, dried methanol/toluene extracts were resuspended using 300 µL of dimethyl sulfoxide (DMSO) and dried hexane extracts were resuspended using 200 µL of DMSO.

Nasal epithelial cell viability. Human primary nasal epithelial cells (Celprogen Inc., Torrance, CA, USA) were grown in human nasal primary cell culture complete medium with serum in a humidified incubator with 95% air and 5% CO_2 at 37 °C. Human nasal cells were seeded in 96-well plates (2000-4000 cells/well, Thermo Fisher Scientific, Waltham, MA) and grown to 60-70% confluence. The culture medium was then removed and the nasal cells were treated with 100 µL of exposure medium from field blanks or samples, each in triplicate for 48 hrs. The exposure medium was composed of 100% fresh cell culture medium (control, 0%), a mixture of fresh cell culture medium and aqueous $PM_{2.5}$ extracts (2.5-20%, v:v), or a mixture of fresh cell culture medium and dimethylsulfoxide (DMSO, 0.25-1.25%, v:v) containing the suspended

organic solvent extracts of $PM_{2.5}$ (either from methanol/toluene, or hexane; see SI methods for details). After 48 hrs, cytotoxicity was determined using a cell counting kit (CCK-8; Dojindo Laboratories, Kumamoto, Japan). Briefly, after 10 µL of CCK-8 solution was added to each well, the nasal cells were incubated at 37 °C for 2 hrs and the optical density (OD) of each well was measured at 450 nm using a microplate reader (BioTek Instruments, Inc., Winooski, VT, USA). Viability of the cells was calculated according to the following equation [1]:

$$\text{Cell viability (\%)} = \frac{(\text{OD value of treatment} - \text{OD value of blank control})}{(\text{OD value of control} - \text{OD value of blank control})} \times 100\%$$

The toxicity (%) of the human nasal cells was determined by the equation: toxicity (%) = 100% - cell viability (%).

Statistical Modelling. Before statistical modelling, all variables (i.e., LC-HRMS features, metals, ions, and gas concentrations) were unit-variance (UV) scaled and divided by the volume of air sampled (except for the gas concentrations). Principal components analysis (PCA), and orthogonal partial least squares discriminant analysis (OPLS-DA) were performed using SIMCA 17 (Sartorius Stedim Data Analytics AB, Sweden). T-distributed stochastic neighbor embedding (t-sne) analysis was performed using the “Rtne” package for R [32]. After parameter optimizations, perplexity of 2 and 2000 steps were selected for the t-sne analysis.

Supplementary results

Back-Trajectories and Ambient Conditions Over the Study Period. The geographic origins of sampled air were evaluated hourly by 48 hr back-trajectories, and in a clustering analysis these were broadly grouped into six source regions (Figure 1A). We classify source region 1 (East Sea/Sea of Japan) and region 2 (East China Sea) as maritime regions, due to their primary source regions over the sea. Region 3 was primarily from the Korean Peninsula, while region 4 primarily originated in north-eastern China, including Shandong Province but also extending into Beijing. Source regions 5 and 6 included air travelling longer-range from northern China and Mongolia. Although these two source regions appear geographically similar, air coming from region 6 moves faster and remains at higher altitudes (>2 km) compared to the air coming from region 5. Taking into account the transport speed, altitude, and timing of descent into the near-surface layer (which is relevant when considering chemical emissions), regions 5 and 6 were selected as two distinct back-trajectory cluster regions. Given the duration of each sample collection period (48-72 hrs), most samples had contributions from more than one geographical region (Figure 1B), although some particularly stable periods were evident, for example, in late spring and summer 2020, when the air was primarily or exclusively from one of the maritime regions.

Over the whole 16-month sampling period, the air coming from the Korean peninsula (region 3) was the main individual source region, contributing to 22% of sampled air. However, the cumulative contribution of air coming from northern China (regions 4, 5, and 6) was 46%, approaching half of all sampled air. This relative distribution of air masses reaching the observatory was similar to what Kim *et al.* (2018) reported between 2008 and 2018, whereby a large contribution of air also came from north China [2], particularly in winter months as observed here (Figure 1B).

Concentrations of $PM_{2.5}$ measured gravimetrically on the analyzed filters ranged from 4.9 to 39.2 µg/m^3 (median 15.9 µg/m^3, Figure 1C) and was correlated with the independently measured AQMS $PM_{2.5}$ during the same period (Pearson's R = 0.67, p = 5.5E^{-12}). The highest concentrations of $PM_{2.5}$ were measured during winter months (Figure 1C), which is a similar result to Kim *et al.* (2020) who observed highest $PM_{2.5}$ concentrations on Jeju Island in both winter and spring months between 2015 and 2019 [3]. The $PM_{2.5}$ concentrations started to decline in our sampling period in late February 2020 (Figure 1C), and in general the $PM_{2.5}$ concentrations during the current campaign are lower than what was previously reported from Jeju Island [3], likely due to the lockdowns and slowed economical activities locally and in surrounding countries due to COVID-19 [4]. Indeed, Kim et al. observed $PM_{2.5}$ concentrations that were always higher than 20 µg/m^3 in spring and winter, and always higher than 15 µg/m^3 in fall and winter [3] which was not the case here. Nevertheless, it is noteworthy that throughout the sampling period at this remote site in the Yellow Sea, 22% of samples (i.e. 19 out of 85) exceeded the 2021 WHO air quality guideline limit of 15 µg/m^3 (Figure 1C).

Hourly ambient concentrations of $PM_{2.5}$, PM_{10}, SO_2, CO, O_3 and NO_2 were monitored at the observatory throughout the campaign (Table 1). SO_2 levels were similar to what Kim *et al.* (2013) measured in 2008 at the same site, with mean SO_2 concentrations of 9.54 (±3.08) ppb during a polluted period [5]. However, PM_{10} concentrations were lower than reported by Kim *et al.* (mean 87.2 ± 21.1 µg/m^3 during the polluted period and mean 30.8 ± 3.82 µg/m^3 during a cleaner period [5]), likely due to slowed economics activities as discussed above. NO_2 and O_3 levels measured in the present study are in the same range as reported by Han *et al.* (2017) at this site [6]; e.g. NO_2 2-14 ppb, O_3 30-80 ppb reported here while Han *et al.* measured NO_2 between 2 and 14 ppb and O_3 between 20 and 90 ppb. CO concentrations in the current campaign (80-330 ppb, Figure S1-C) were also comparable to Kim *et al.* [7] who reported concentrations of 150 - 300 ppb between January 2017 and November 2018 at the same site.

Cell viability assay. Prior to performing chemical analyses, the human nasal cell viability was measured with treatment of water, methanol/toluene, and hexane extracts that included, respectively, water soluble components, polar compounds, and non-polar compounds. The results of the nasal cell viability are summarized in Figure S2. Since 48hr-treatment with 20% of aqueous solution (a mixture of 80% fresh cell culture medium and 20% water) to the nasal cells did not affect the viability of the nasal cells (data not shown), different percentages of aqueous $PM_{2.5}$ extracts, up to 20%, were tested in the cell viability assay. As shown in Figure S2A, water extracts from two of the three filters (2019.10.25 and 2019.11.11) showed distinct toxicity whereas the toxic effect from the field blank was negligible. As for the methanol/toluene, and hexane extracts, the dried extracts were resuspended using DMSO to solubilize the extracted components for exploring the toxic effects of the extracted components. Due to the toxic effects of DMSO itself, the tested percentages of the extracts for the human nasal cells were limited to 1.25%. As can be seen in Figure S2B and S2C, the toxic effects of the extracts of polar and non-polar components from the three sample filters in comparison to the filed blank were not observed. Both organic extracts were pre-concentrated (unlike the water extracts) meaning that the proportion of actual filter the cells were exposed to was higher for these extracts than for the water extracts. When the actual doses exposed to the nasal cells were calculated, 20% of water extracts in the media corresponded to exposure of 20 µL out a total of 20 mL of water extracts, which means that the actual dose of water-soluble components from 1/4 piece of $PM_{2.5}$ sample filter was 0.1%. On the other hand, 1.25% of methanol/toluene and hexane extracts corresponded to approximately 0.42% and 0.625% of the methanol/toluene and hexane extracts, respectively. This means that even without pre-concentration the water-soluble components induced more cytotoxic effects on the human nasal cells in comparison to the polar and non-polar components.

Metals and Major Ions in Aqueous $PM_{2.5}$ Extracts. Median concentrations and ranges for 11 major ions and 17 trace metals or metalloids in aqueous extracts of all $PM_{2.5}$ samples are in Table 1 and Figure S1. Sulphate (SO_4^{2-}), nitrate (NO_3^-), ammonium (NH_4^+) and sodium (Na^+) were the most abundant major ions, as also observed by Lim *et al.* (2012) in water-extracted $PM_{2.5}$ at the same site [8]. The major detectable metals were iron, zinc, aluminium, titanium, manganese and lead. $PM_{2.5}$ water-soluble zinc and lead have previously been linked with

industrial activities in major Chinese cities [9], and iron, zinc, and aluminium were also the most abundant metals measured in Changzhou (industrial area in China) in 2016 autumn and winter [10]. The Spearman correlation matrix (Table S7) between all metals and transition elements measured by ICP-MS showed many strong correlation coefficients (> 0.7), suggesting common sources and similar trends during the campaign. For example, Zn and Pb had a Spearman coefficient of 0.82, and these have been reported to have similar airborne sources in Beijing, from coal combustion and vehicle exhaust in autumn and winter, and an increasing industrial contribution in spring and summer [11].

Supplementary figures

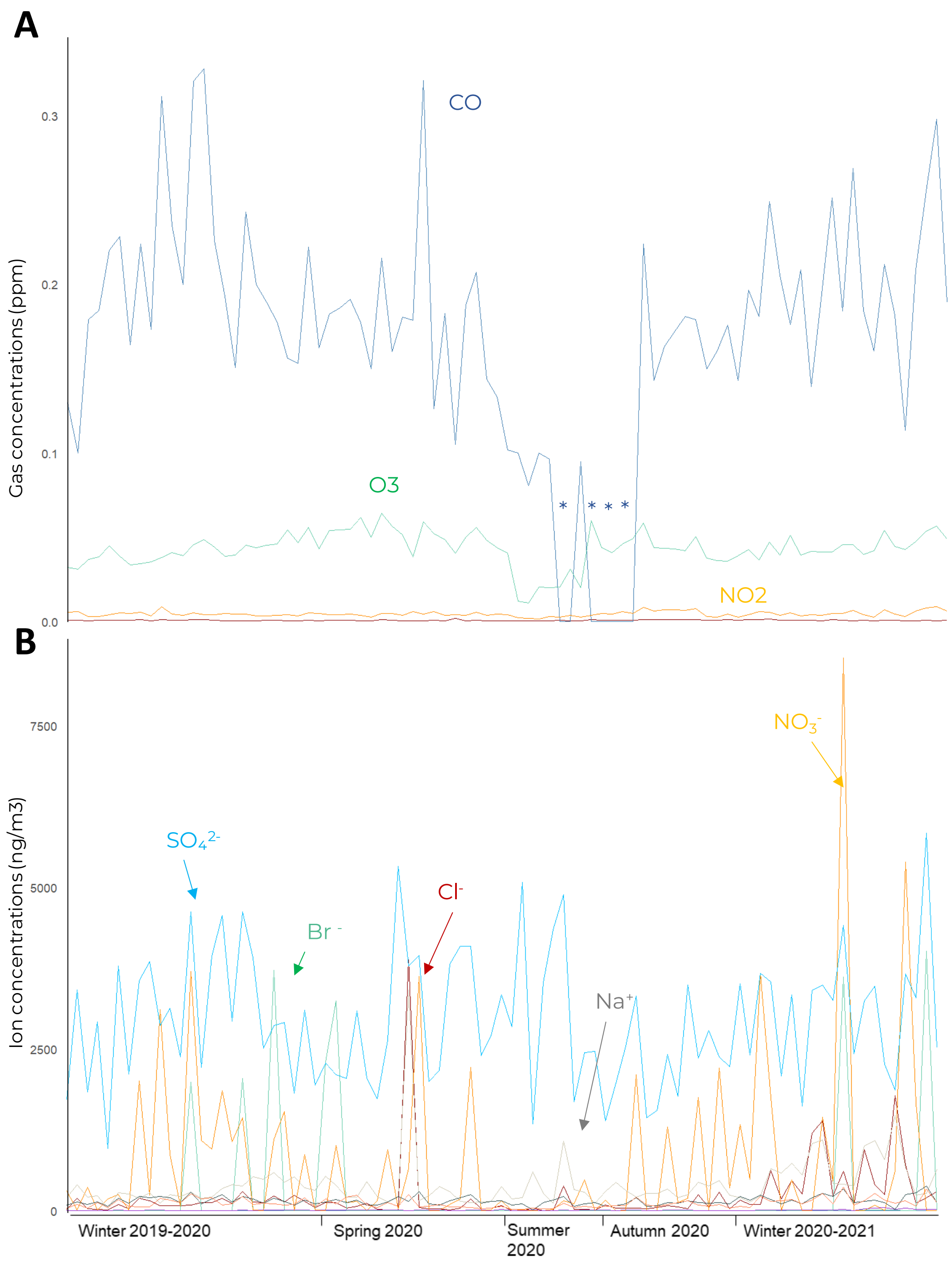

*Figure S1: A) Gas concentrations (in ppm) measured at the sampling station during the sampling campaign. *=missing data points. B) Concentrations of the major ions measured in the water-extracted $PM_{2.5}$.*

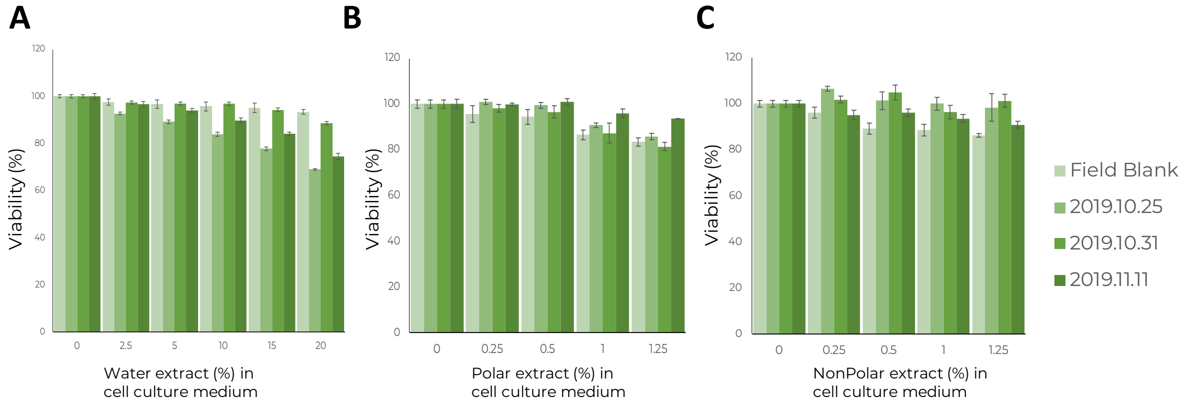

Figure S2: Human nasal cellular viability for water and solvent extracts of three $PM_{2.5}$ samples and a field blank. A) Water extracts dosed between 0 and 20% of total assay volume. B) Pre-concentrated polar extracts representing between 0 and 1.25% of the assay's volume. B) Pre-concentrated nonpolar extracts representing between 0 and 1.25% of the assay's volume.

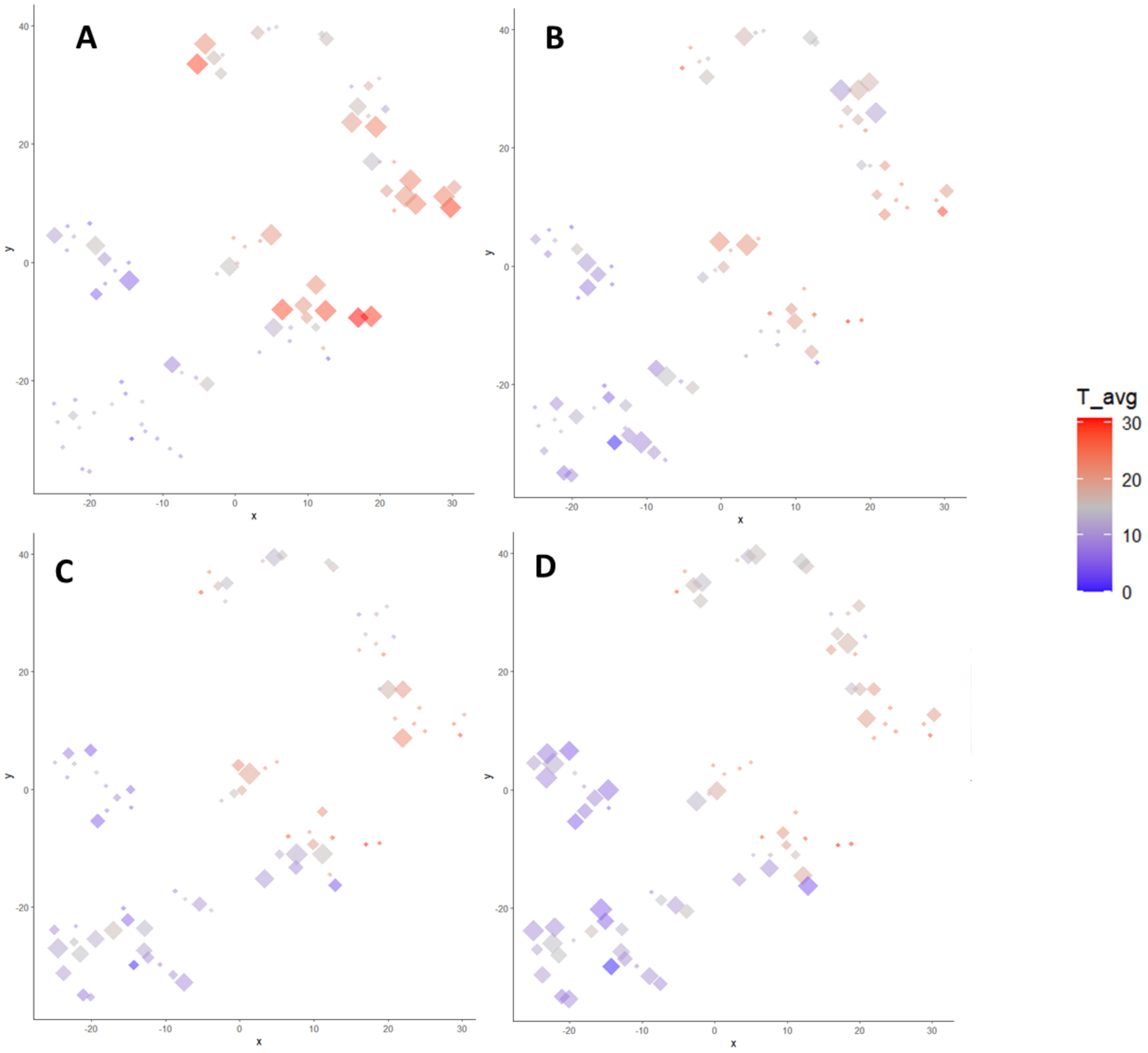

Figure S3: Results of t-sne analysis with each sample coloured by average temperature during collection, but sized according to increased influence of the back-trajectory source region: (A) maritime regions (cluster 1 + cluster 2), (B) Korean peninsula (cluster 3), (C) Shandong/Beijing (cluster 4), and (D) North China/Mongolia (cluster 5 + cluster 6). Smaller data points indicate samples least influenced by the selected cluster, larger data points indicate samples more influenced by the selected cluster.

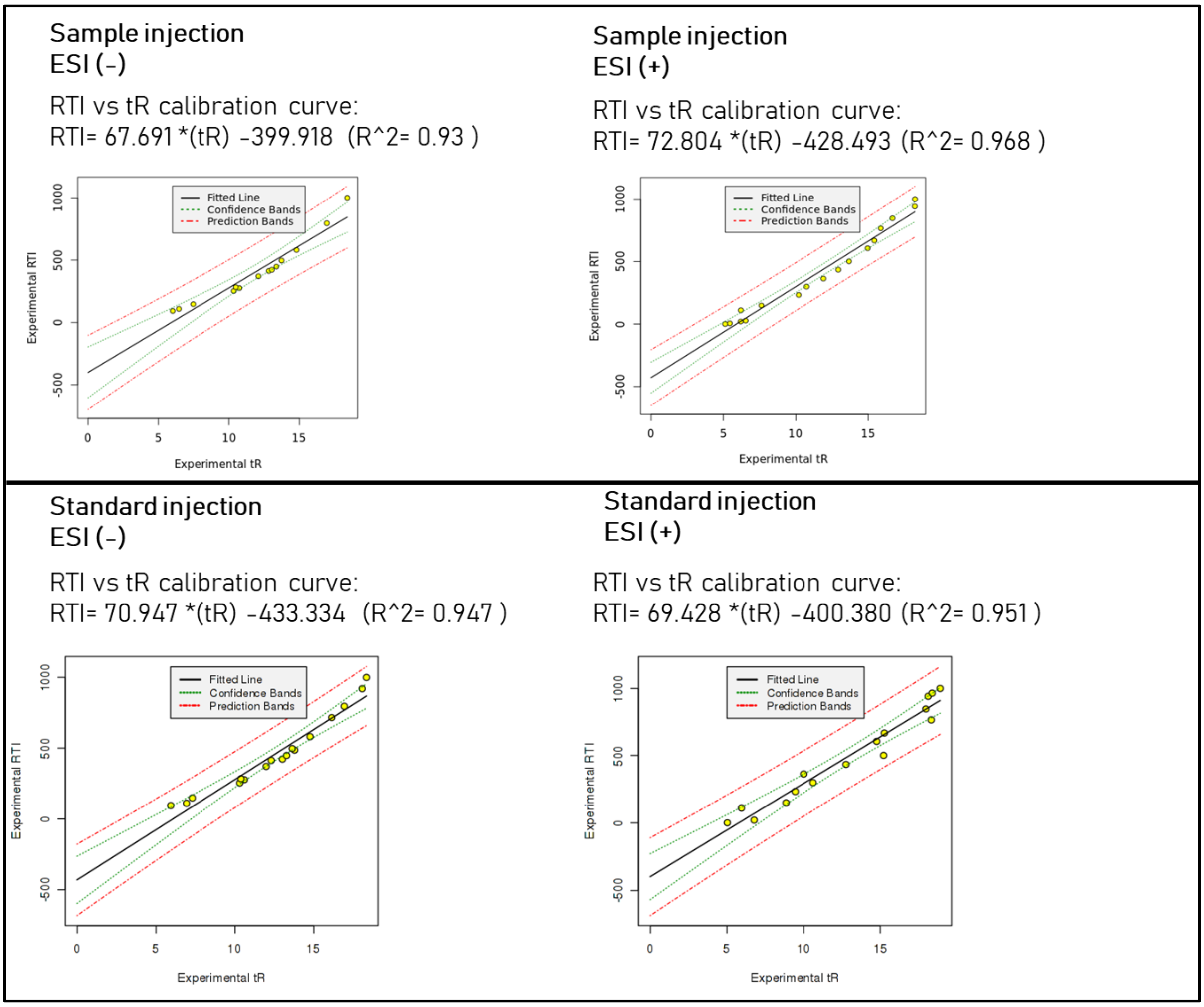

Figure S4: Retention time index (RTI) calibration curves and equations for the sample injection (measured during the injection of all the samples and field blanks over seven days using the aligned retention times of the RTI standards solution injected every day, top panel) and the standard injection (measured during the injection of reference standards for analytical confirmation of annotated compounds, bottom panel) in positive (ESI +) and negative (ESI -) ionization modes. tR = experimental retention time.

	WSOC_neg_450
Substance	Orcinol (C7H8O2)
Precursor ion (accurate mass)	$[M\text{-}H]^-$ = 123.04526<br>Δm/z = 0.65 ppm
Retention time	ΔRTI =24.59
Isotopic fit	✓
Most intense **experimental** fragment ion	✓
All other **experimental** fragment ions	✓
Acquired mode	DIA in sample, DDA from standard
**Confidence level (Schymanski et al., 2014)**	**1 (confirmed by standard)**

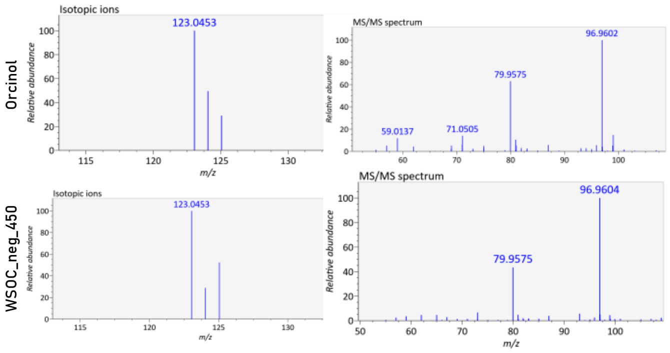

Figure S5: Comparison between feature WSOC_neg_450 detected in the samples and orcinol monohydrate's standard injection.

	WSOC_neg_3709
Substance	3-Phenoxybenzoic acid ($C13H10O3$)
Precursor ion (accurate mass)	$[M\text{-}H]^-$ = 213.05589<br>Δm/z = 4.18 ppm
Retention time	ΔRTI =24.39
Isotopic fit	✓
Most intense **experimental** fragment ion	✓
All other **experimental** fragment ions	✓
Acquired mode	DIA in sample, DDA from standard
**Confidence level (Schymanski et al., 2014)**	**1 (confirmed by standard)**

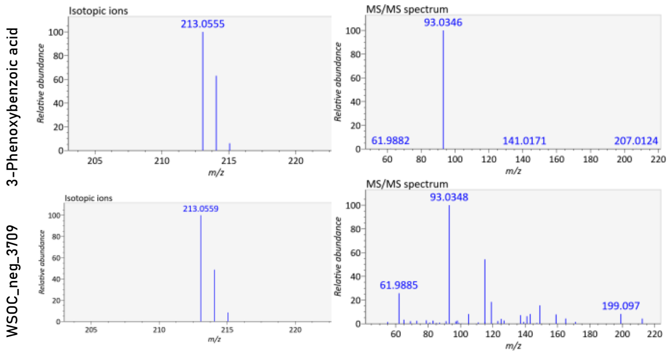

Figure S6: Comparison between feature WSOC_neg_3709 detected in the samples and 3-Phenoxybenzoic acid's standard injection.

	**WSOC_neg_5383**
Substance	Pentadecanoic acid (C15H30O2)
Precursor ion (accurate mass)	$[M\text{-}H]^{-}$ = 241.21765<br>Δm/z = 0.95 ppm
Retention time	ΔRTI =11.93
Isotopic fit	✓
Most intense **experimental** fragment ion	✓
All other **experimental** fragment ions	✓
Acquired mode	DIA in sample, DDA from standard
**Confidence level (Schymanski et al., 2014)**	**1 (confirmed by standard)**

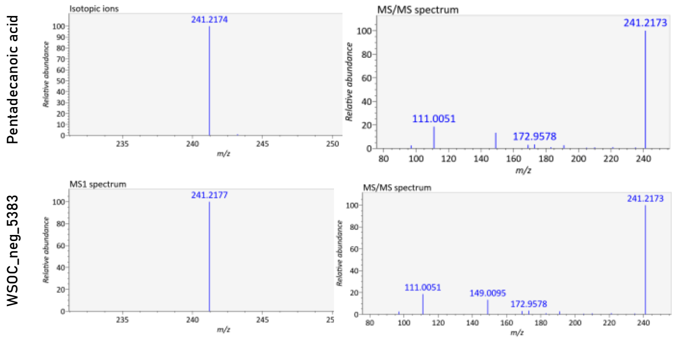

Figure S7: Comparison between feature WSOC_neg_5383 detected in the samples and Pentadecanoic acid's standard injection.

	WSOC_neg_18543
Substance	Perfluorononanoic acid (C9HF17O2)
Precursor ion (accurate mass)	[M-H]- = 462.96368<br>Δm/z = 0.73 ppm
Retention time	ΔRTI =8.72
Isotopic fit	✓
Most intense **experimental** fragment ion	✓
All other **experimental** fragment ions	✓
Acquired mode	DIA in sample, DDA from standard
**Confidence level (Schymanski et al., 2014)**	**1 (confirmed by standard)**

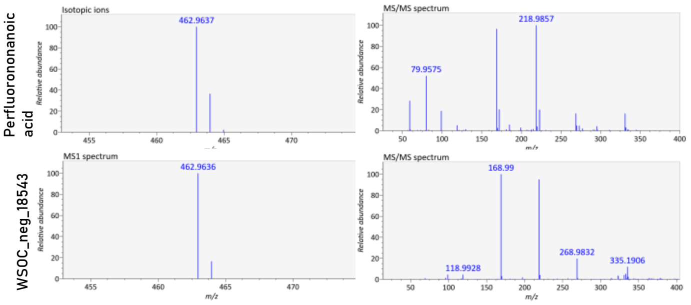

Figure S8: Comparison between feature WSOC_neg_18543 detected in the samples and Perfluorononanoic acid's standard injection.

	**WSOC_neg_16301**
Substance	Perfluorooctanoic acid (C8HF15O2)
Precursor ion (accurate mass)	$[M-H]^-$ = 412.96676<br>Δm/z = 0.31 ppm
Retention time	ΔRTI =10.85
Isotopic fit	✓
Most intense **experimental** fragment ion	✓
All other **experimental** fragment ions	✓
Acquired mode	DIA in sample, DDA from standard
**Confidence level (Schymanski et al., 2014)**	**1 (confirmed by standard)**

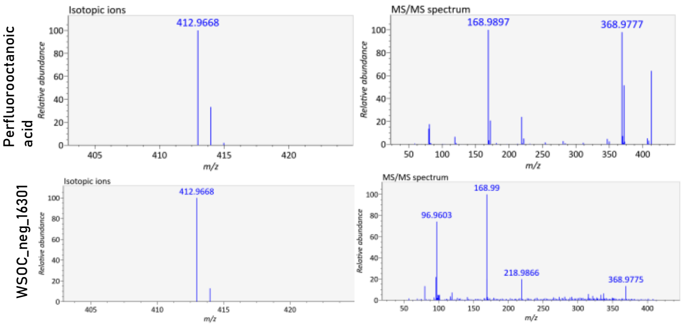

Figure S9: Comparison between feature WSOC_neg_16301 detected in the samples and perfluorooctanoic acid's standard injection.

	WSOC_pos_1058
Substance	α-Pinene
Precursor ion (accurate mass)	$[M+H]^+$ = 135.11694<br>Δm/z = 0.44 ppm
Retention time	ΔRTI =25.06
Isotopic fit	✓
Most intense **experimental** fragment ion	✓
All other **experimental** fragment ions	✓
Acquired mode	DIA in sample, DDA from standard
**Confidence level (Schymanski et al., 2014)**	**1 (confirmed by standard)**

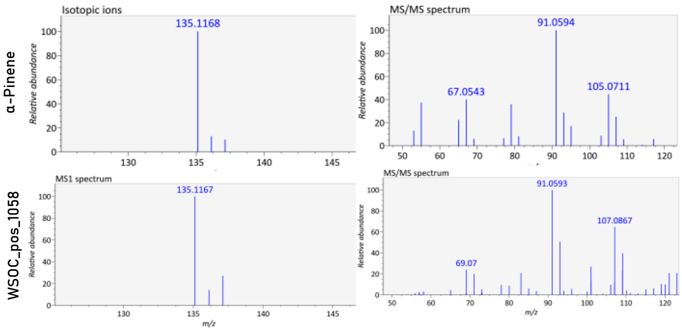

Figure S10: Comparison between feature WSOC_pos_1058 detected in the samples and α-Pinene's standard injection.

	**WSOC_pos_2179**
Substance	(−)-Nicotine
Precursor ion (accurate mass)	$[M+H]^+$ = 163.13298<br>Δm/z = 0.37 ppm
Retention time	ΔRTI =27.63
Isotopic fit	✓
Most intense **experimental** fragment ion	✓
All other **experimental** fragment ions	✓
Acquired mode	DIA in sample, DDA from standard
**Confidence level (Schymanski et al., 2014)**	**1 (confirmed by standard)**

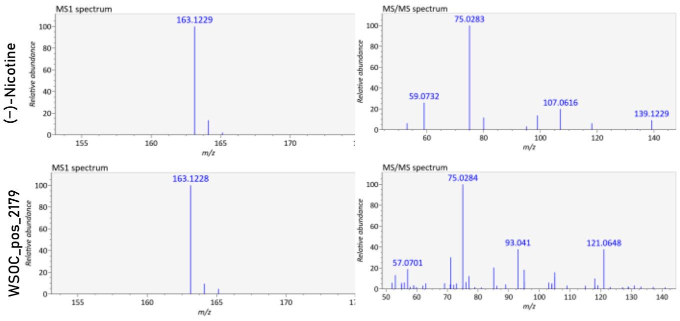

Figure S11: Comparison between feature WSOC_pos_2179 detected in the samples and (-)-Nicotine's standard injection.

	WSOC_pos_6651
Substance	Bentazone
Precursor ion (accurate mass)	$[M+H]^+$ = 241.08607<br>Δm/z = 2.45 ppm
Retention time	ΔRTI = 1.56
Isotopic fit	✓
Most intense **experimental** fragment ion	✓
All other **experimental** fragment ions	✓
Acquired mode	DIA in sample, DDA from standard
**Confidence level (Schymanski et al., 2014)**	**1 (confirmed by standard)**

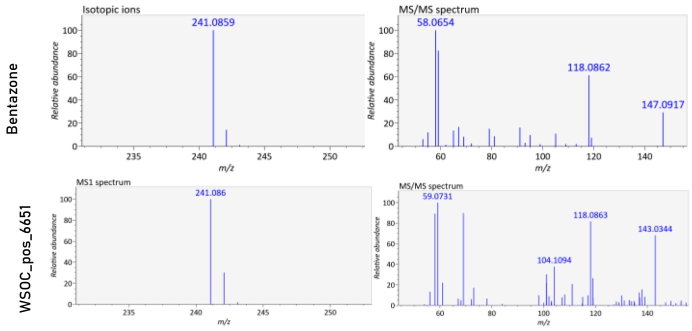

Figure S12: Comparison between feature WSOC_pos_6651 detected in the samples and Bentazone's standard injection.

	WSOC_pos_7824
Substance	Dibutyl adipate
Precursor ion (accurate mass)	$[M+H]^+$ = 259.19078<br>Δm/z = 2.01 ppm
Retention time	ΔRTI = 8.93
Isotopic fit	✓
Most intense **experimental** fragment ion	✓
All other **experimental** fragment ions	✓
Acquired mode	DIA in sample, DDA from standard
**Confidence level (Schymanski et al., 2014)**	**1 (confirmed by standard)**

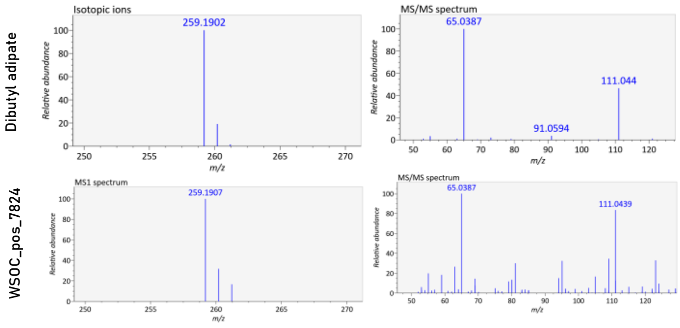

Figure S13: Comparison between feature WSOC_pos_7824 detected in the samples and Dibutyl adipate's standard injection.

	**WSOC_pos_6033**
Substance	Dehydrocostus lactone ($C15H18O2$)
Precursor ion (accurate mass)	$[M+H]^+$ = 231.13481<br>Δm/z = 1.69 ppm
Retention time	ΔRTI = 5.97
Isotopic fit	✓
Most intense **experimental** fragment ion	✓
All other **experimental** fragment ions	✓
Acquired mode	DIA in sample, DDA from standard
**Confidence level (Schymanski et al., 2014)**	**1 (confirmed by standard)**

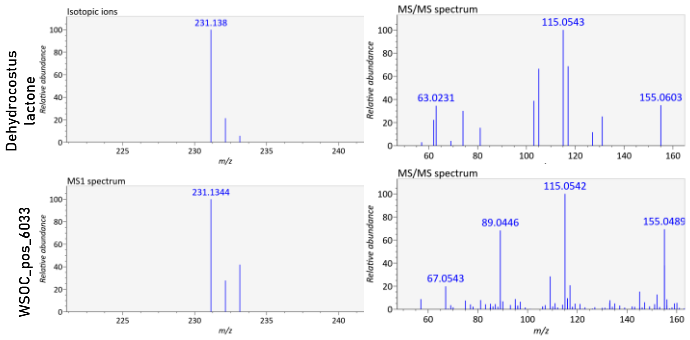

Figure S14: Comparison between feature WSOC_pos_6033 detected in the samples and Dehydrocostus lactone's standard injection.

	WSOC_neg_2601
Substance	1-Naphthalenesulfonic acid
Precursor ion (accurate mass)	$[M\text{-}H]^-$ = 207.01228<br>Δm/z = 0.87 ppm
Retention time	ΔRTI = 21.44
Isotopic fit	✓
Most intense **experimental** fragment ion	✓
All other **experimental** fragment ions	✓
Acquired mode	DIA in sample, DDA from standard
**Confidence level (Schymanski et al., 2014)**	**1 (confirmed by standard)**

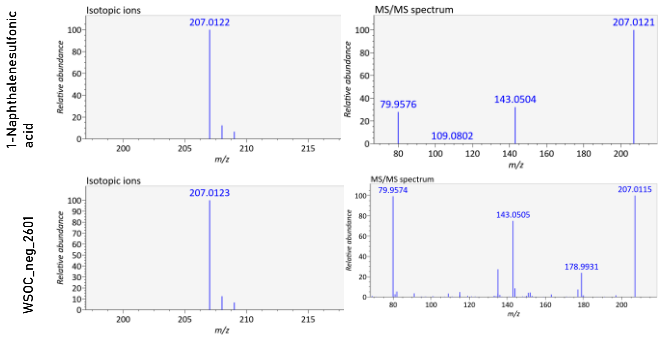

Figure S15: Comparison between feature WSOC_neg_2601 detected in the samples and 1-Naphthalenesulfonic acid's standard injection.

	WSOC_neg_14887
Substance	Salazinic acid
Precursor ion (accurate mass)	$[M-H]^-$ = 387.03575<br>Δm/z = 1.42 ppm
Retention time	ΔRTI = 17.88
Isotopic fit	✓
Most intense **experimental** fragment ion	✓
All other **experimental** fragment ions	✓
Acquired mode	DIA in sample, DDA from standard
**Confidence level (Schymanski et al., 2014)**	**1 (confirmed by standard)**

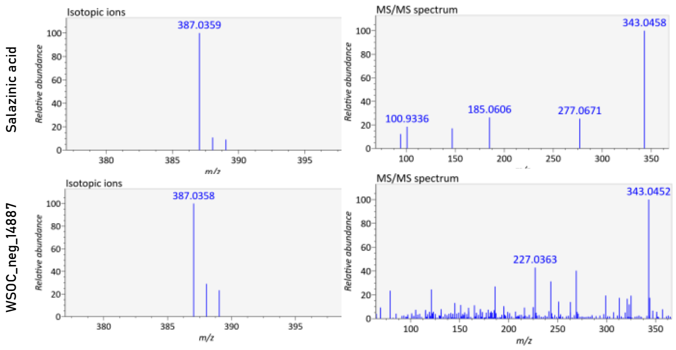

Figure S16: Comparison between feature WSOC_neg_14887 detected in the samples and Salazinic acid's standard injection.

	WSOC_neg_2350
Substance	Azelaic acid
Precursor ion (accurate mass)	$[M-H]^-$ = 187.0978<br>Δm/z = 0.43 ppm
Retention time	ΔRTI = 21.68
Isotopic fit	✓
Most intense **experimental** fragment ion	✓
All other **experimental** fragment ions	✓
Acquired mode	DIA in sample, DDA from standard
**Confidence level (Schymanski et al., 2014)**	**1 (confirmed by standard)**

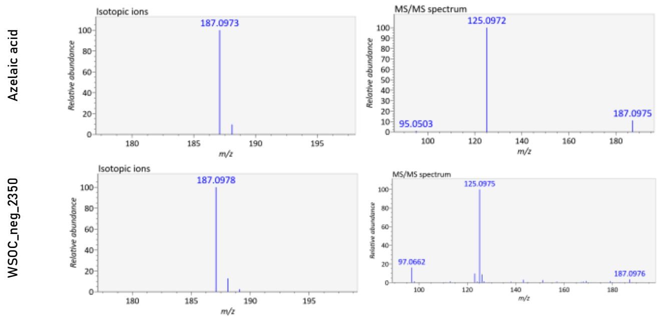

Figure S17: Comparison between feature WSOC_neg_2350 detected in the samples and Azelaic acid's standard injection.

	WSOC_pos_2391
Substance	3,4-Dimethoxybenzaldehyde
Precursor ion (accurate mass)	$[M+H]^+$ = 167.07032<br>Δm/z = 0.12 ppm
Retention time	ΔRTI = 21.75
Isotopic fit	✓
Most intense **experimental** fragment ion	✓
All other **experimental** fragment ions	✓
Acquired mode	DIA in sample, DDA from standard
**Confidence level (Schymanski et al., 2014)**	**1 (confirmed by standard)**

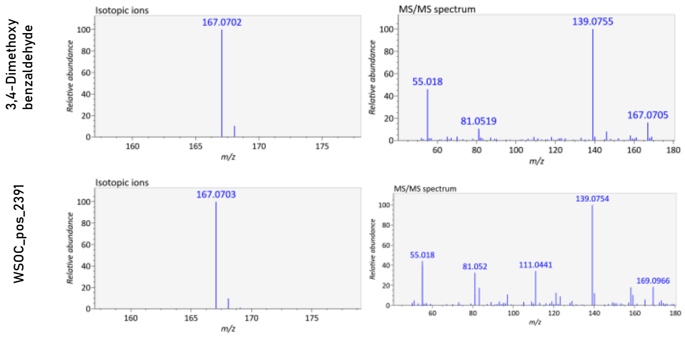

Figure S18: Comparison between feature WSOC_neg_2391 detected in the samples and 3,4-Dimethoxybenzaldehyde's standard injection.

	WSOC_neg_758
Substance	Salicylic acid
Precursor ion (accurate mass)	$[M-H]^-$ = 137.02453<br>Δm/z = 0.36 ppm
Retention time	ΔRTI = 7.63
Isotopic fit	✓
Most intense **experimental** fragment ion	✓
All other **experimental** fragment ions	✓
Acquired mode	DIA in sample, DDA from standard
**Confidence level (Schymanski et al., 2014)**	**1 (confirmed by standard)**

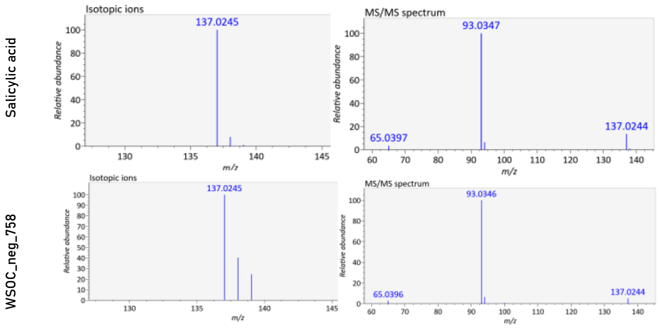

Figure S19: Comparison between feature WSOC_neg_758 detected in the samples and Salicylic acid's standard injection.

	WSOC_pos_8676
Substance	N-Dodecanoyl-N-methylglycine
Precursor ion (accurate mass)	$[M+H]^+$ = 272.22186<br>Δm/z = 0.44 ppm
Retention time	ΔRTI = 8.27
Isotopic fit	✓
Most intense **experimental** fragment ion	✓
All other **experimental** fragment ions	✓
Acquired mode	DIA in sample, DDA from standard
**Confidence level (Schymanski et al., 2014)**	**1 (confirmed by standard)**

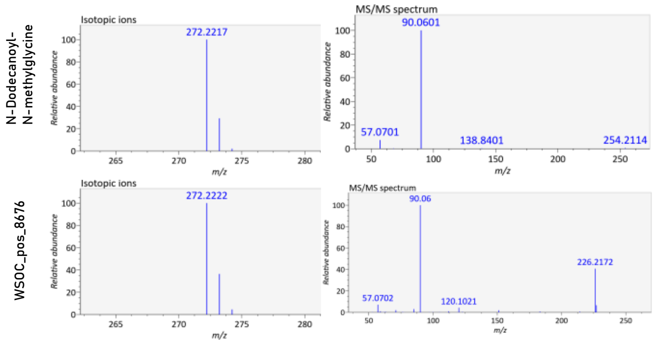

Figure S20: Comparison between feature WSOC_pos_8676 detected in the samples and N-Dodecanoyl-N-methylglycine's standard injection.

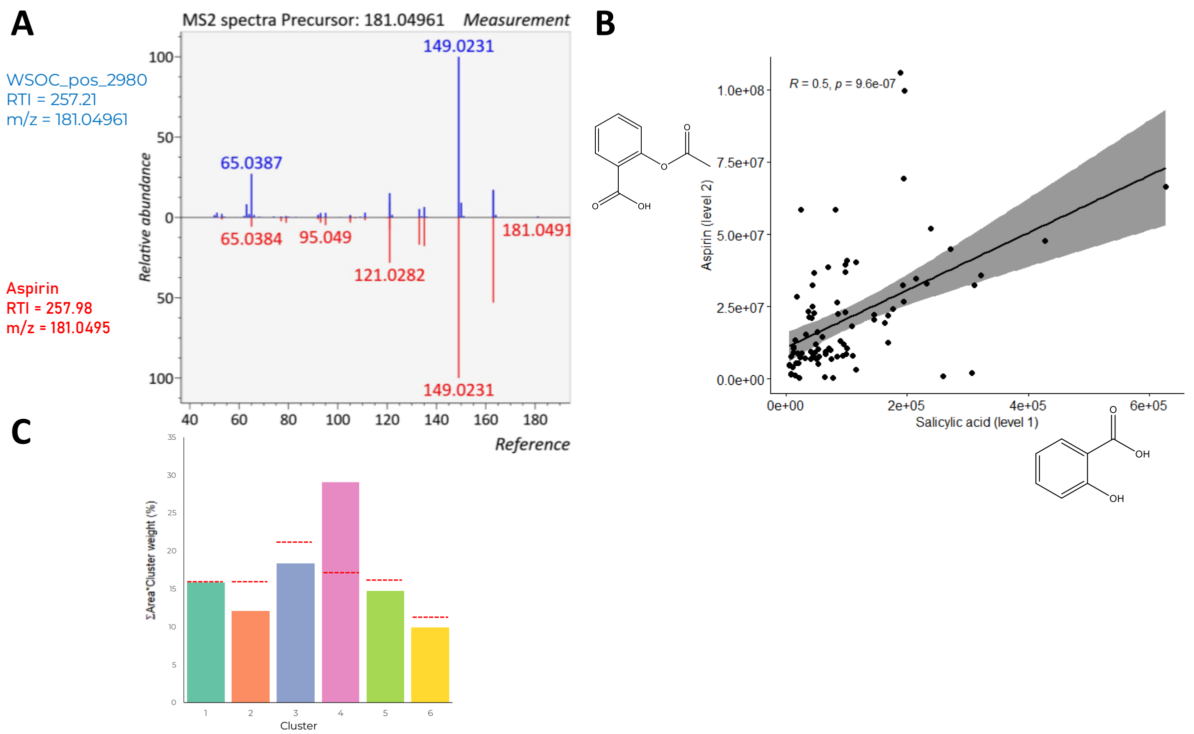

Figure S21: Library match of aspirin with feature WSOC_pos_3456 (A), pearson's correlation between aspirin and salicylic acid (B), and source of geographic regions of the peak annotated as aspirin (C, red dashed lines = contribution of a region to the overall air masses during the sampling campaign).

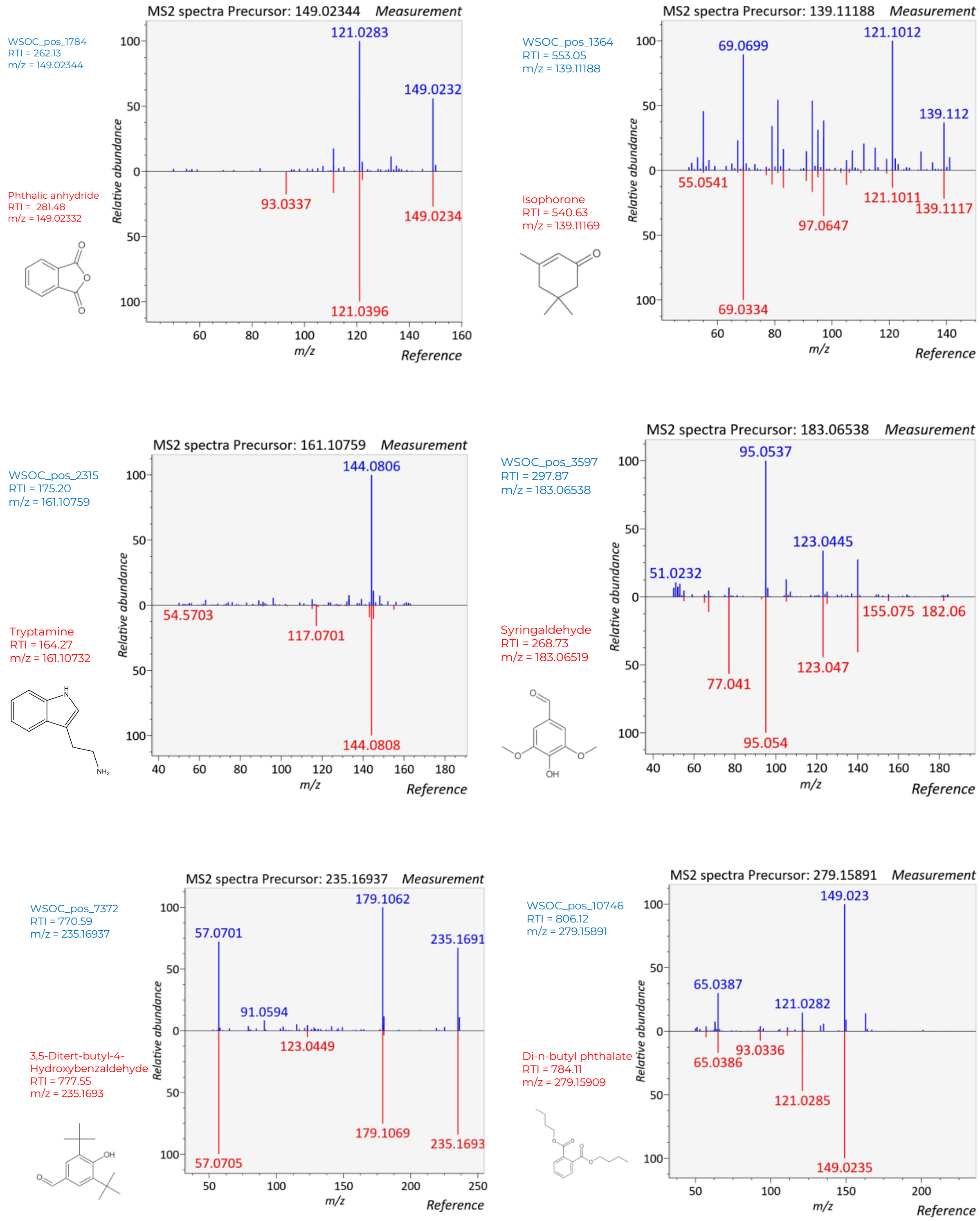

Figure S22: Library match of 6 annotated compounds: phthalic anhydride, Isophorone, tryptamine, syringaldehyde, 3,5-Ditert-butyl-4-hydroxybenzaldehyde, and di-n-butyl phthalate.

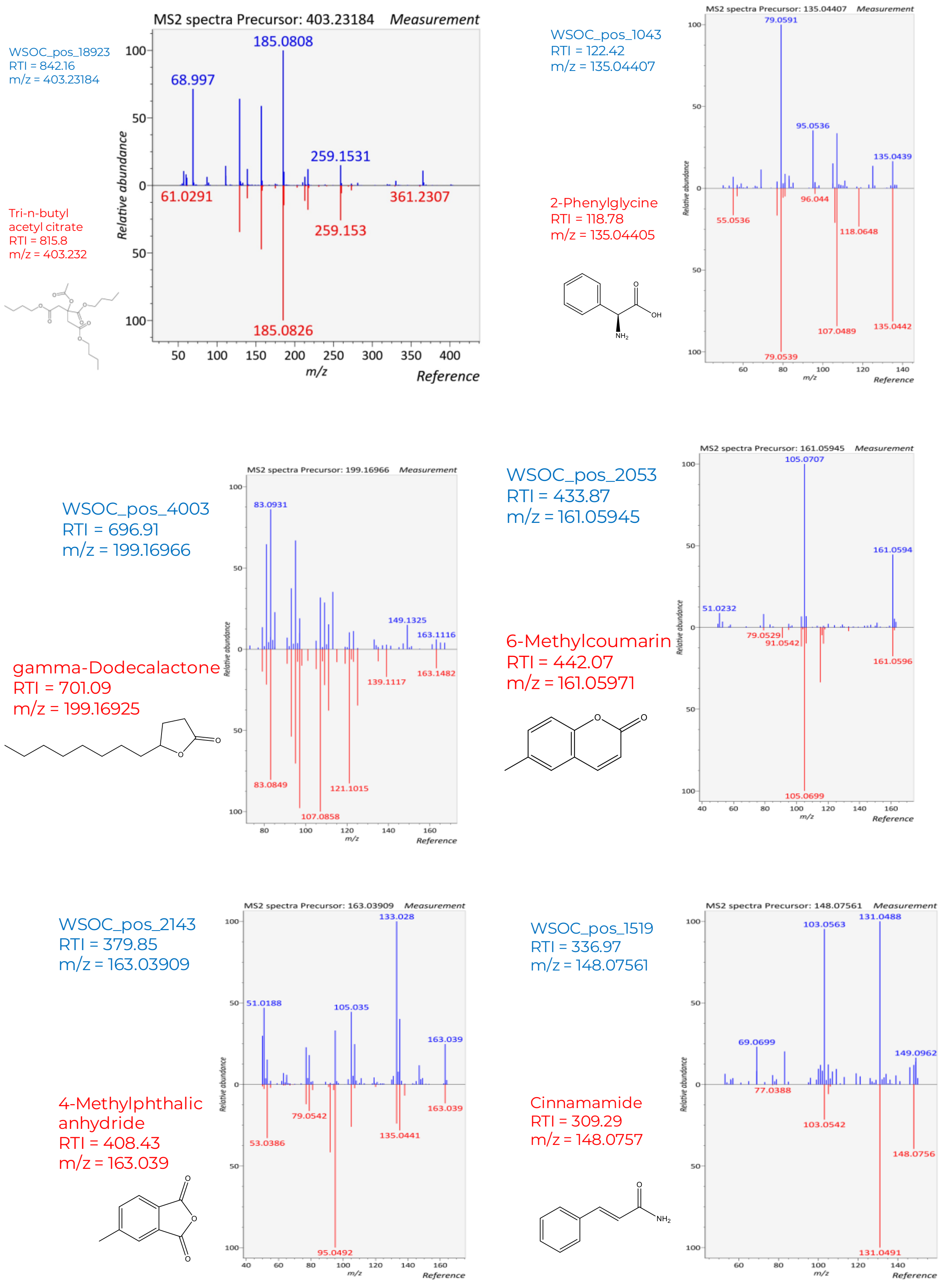

Figure S23: Library match of 6 annotated compounds: tri-n-butyl acetyl citrate, 2-phenylglycine, gamma-dodecalactone, 6-methylcoumarin, 4-methylphthalic anhydride, and cinnamamide

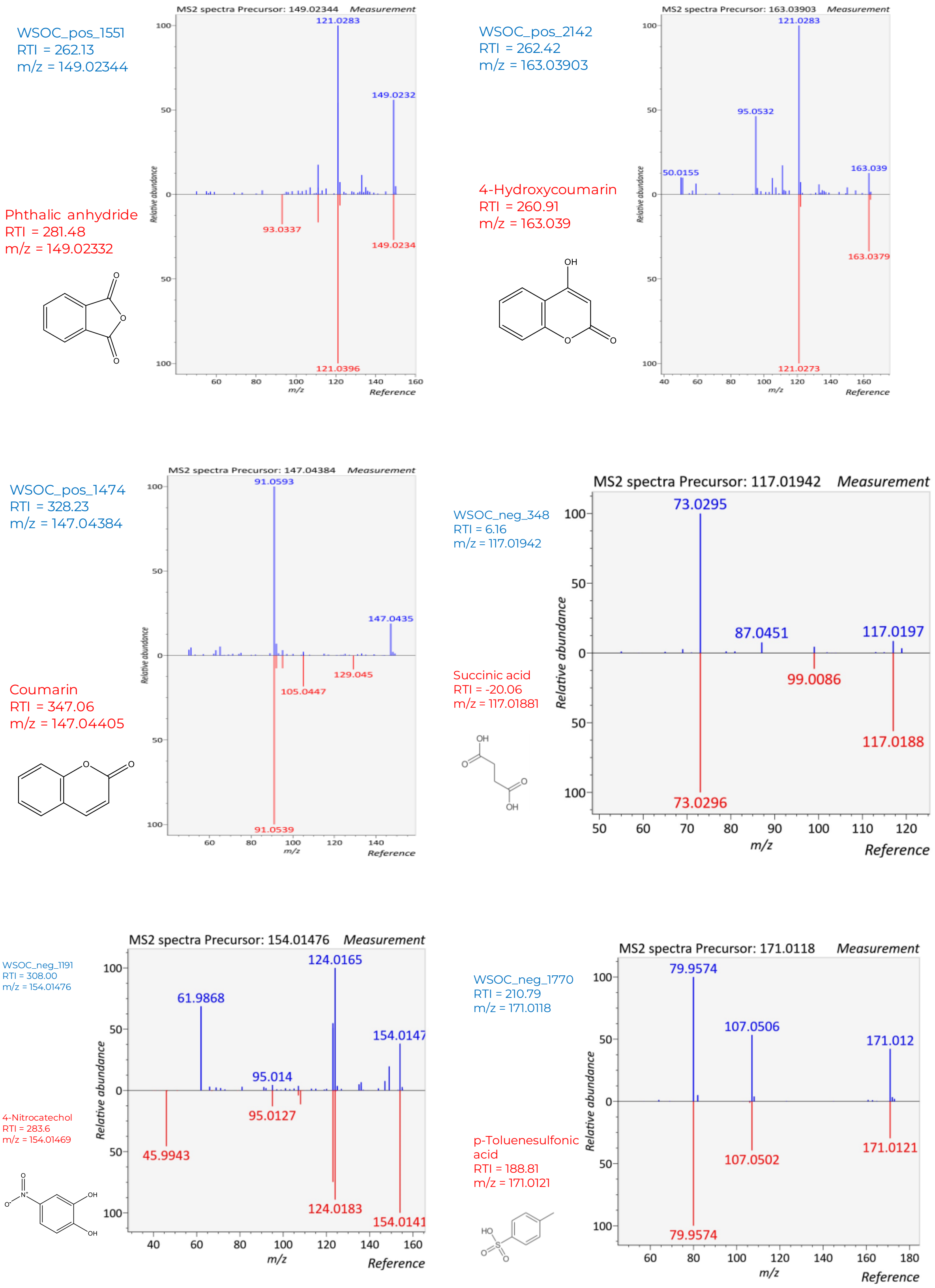

Figure S24: Library match of 6 annotated compounds: phthalic anhydride, 4-hydroxycoumarin, coumarin, succinic acid, 4-nitrocatechol, and p-toluenesulfonic acid

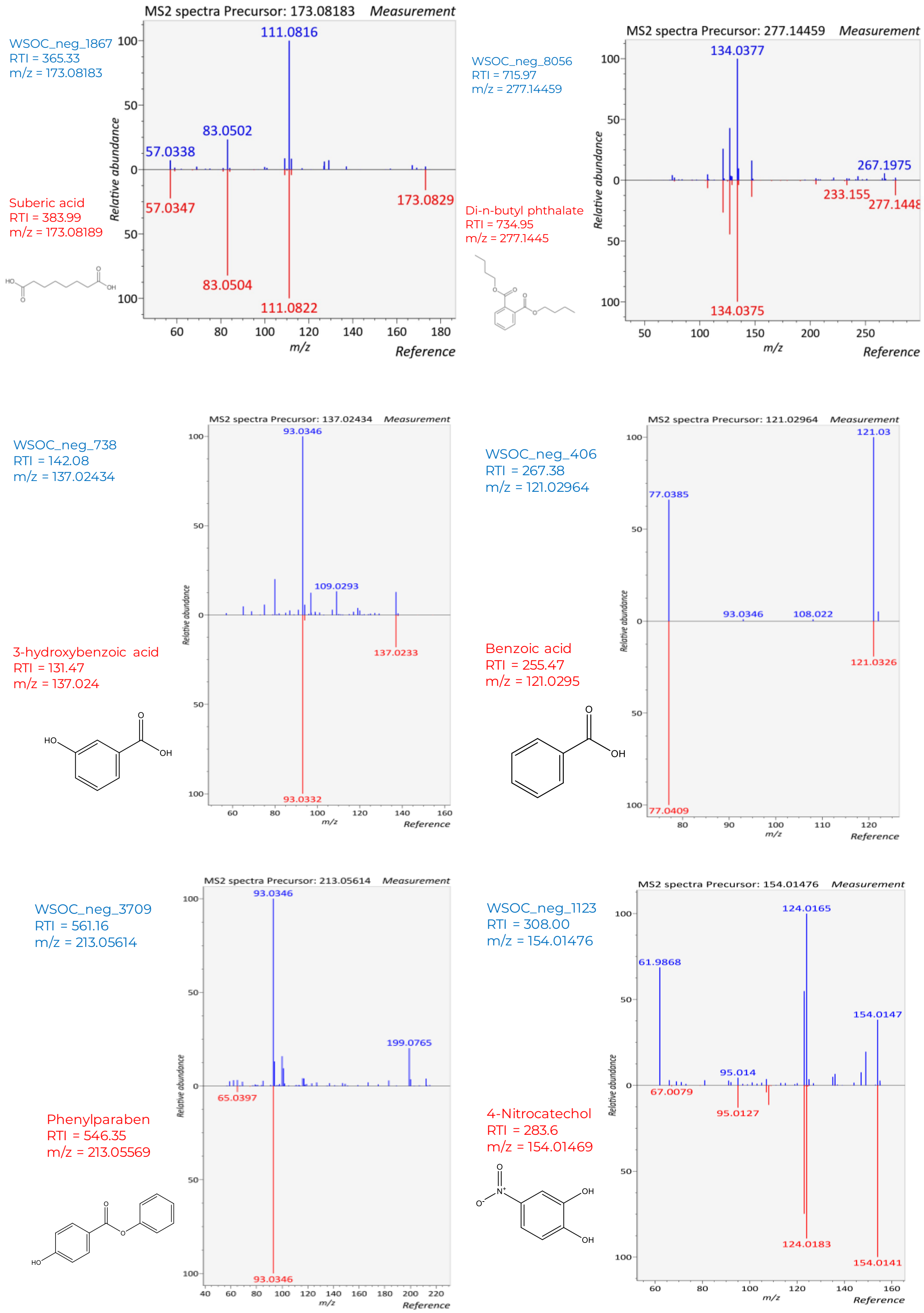

Figure S25: Library match of 6 annotated compounds: suberic acid, di-n-butyl phthalate, 3-hydroxybenzoic acid, benzoic acid, phenylparaben, and 4-nitrocatechol.

REFERENCES

1. Wei, H., W. Yuan, H. Yu, and H. Geng, *Cytotoxicity induced by fine particulate matter (PM 2.5) via mitochondria-mediated apoptosis pathway in rat alveolar macrophages.* Environmental Science and Pollution Research, 2021. 28: p. 25819-25829.
2. Kim, S.-W., J. Heo, and J.-U. Park, *Relationship between submicron particle formation and air mass history observed in the Asian continental outflow at Gosan, Korea, during 2008–2018.* Air Quality, Atmosphere & Health, 2021. 14(2): p. 291-300.DOI: 10.1007/s11869-020-00934-3.
3. Kim, D., H.E. Choi, W.M. Gal, and S. Seo, *Five year trends of particulate matter concentrations in korean regions (2015–2019): When to ventilate?* International Journal of Environmental Research and Public Health, 2020. 17(16): p. 1-15.DOI: 10.3390/ijerph17165764.
4. Hao, X., J. Li, H. Wang, H. Liao, Z. Yin, J. Hu, Y. Wei, and R. Dang, *Long-term health impact of PM2.5 under whole-year COVID-19 lockdown in China.* Environmental Pollution, 2021. 290(March): p. 118118-118118.DOI: 10.1016/j.envpol.2021.118118.
5. Kim, Y., S.W. Kim, S.C. Yoon, M.H. Kim, and K.H. Park, *Aerosol properties and associated regional meteorology during winter pollution event at Gosan climate observatory, Korea.* Atmospheric Environment, 2013. 85(X): p. 9-17.DOI: 10.1016/j.atmosenv.2013.11.041.
6. Han, J., M. Lee, X. Shang, G. Lee, and L.K. Emmons, *Decoupling peroxyacetyl nitrate from ozone in Chinese outflows observed at Gosan Climate Observatory.* Atmos. Chem. Phys., 2017. 17(17): p. 10619-10631.DOI: 10.5194/acp-17-10619-2017.
7. Kim, W.-H., H.-S. Yang, J.-O. Bu, C.-H. Kang, J.-M. Song, and S. Chambers, *Concentration variability of atmospheric radon and gaseous pollutants at background area of Korea between 2017 and 2018.* 2022.
8. Lim, S., M. Lee, G. Lee, S. Kim, S. Yoon, and K. Kang, *Ionic and carbonaceous compositions of PM10, PM2.5 and PM1.0 at Gosan ABC Superstation and their ratios as source signature.* Atmospheric Chemistry and Physics, 2012. 12(4): p. 2007-2024.DOI: 10.5194/acp-12-2007-2012.
9. Cheng, M.C., C.F. You, J. Cao, and Z. Jin, *Spatial and seasonal variability of water-soluble ions in PM 2.5 aerosols in 14 major cities in China.* Atmospheric Environment, 2012. 60: p. 182-192.DOI: 10.1016/j.atmosenv.2012.06.037.
10. Bi, C., Y. Chen, Z. Zhao, Q. Li, Q. Zhou, Z. Ye, and X. Ge, *Characteristics, sources and health risks of toxic species (PCDD/Fs, PAHs and heavy metals) in PM2. 5 during fall and winter in an industrial area.* Chemosphere, 2020. 238: p. 124620.
11. Yang, X., M. Zheng, Y. Liu, C. Yan, J. Liu, J. Liu, and Y. Cheng, *Exploring sources and health risks of metals in Beijing PM2. 5: Insights from long-term online measurements.* Science of The Total Environment, 2022. 814: p. 151954.
